# A gut feeling: Exploring the effects of probiotics on risk-taking behavior using TMS

**DOI:** 10.1016/j.isci.2026.114696

**Published:** 2026-01-14

**Authors:** Aline M. Dantas, Teresa Schuhmann, Elisabeth Brüggen, Peiran Jiao

**Affiliations:** 1 Department of Cognitive Neurosciences, Maastricht University, Maastricht, the Netherlands; 2 Donders Centre for Cognitive Neuroimaging, Nijmegen, the Netherlands; 3 Department of Marketing and Supply Chain Management, Maastricht University, Maastricht, the Netherlands; 4 Maastricht Brain Imaging Centre, Maastricht, the Netherlands; 5 Brightlands Institute for Smart Society (BISS), Heerlen, the Netherlands; 6 Department of Marketing, Tilburg School of Economics and Management, Tilburg University, Tilburg, the Netherlands; 7 Network for Studies on Pension, Aging, and Retirement (NetSPAR), Tilburg, the Netherlands; 8 Department of Finance, Maastricht University, Maastricht, the Netherlands

**Keywords:** Neuroscience, Behavioral neuroscience, Microbiology

## Abstract

This study explores the impact of the gut-brain axis (GBA) on risk-taking through a 4-week, double-blinded, placebo-controlled probiotics protocol and the Maastricht Gambling Task (MGT). We used transcranial magnetic stimulation (TMS) on the ventromedial prefrontal cortex (VMPFC), a region thought to mediate GBA signals in risk-related decisions. Continuous theta burst stimulation (cTBS) targeted the VMPFC, with the superior parietal lobule (SPL) and sham as control conditions, applied before and after the probiotics/placebo intervention. Results show that probiotics significantly increased risky choices and response times in the MGT compared to placebo, without reducing choice optimality. VMPFC stimulation, independent of probiotics intake, also led to riskier choices when controlling for task repetition. The nonsignificant interaction between probiotics and VMPFC stimulation suggests that the VMPFC does not play a key functional role in the effects of probiotics on risk-taking behavior. These findings offer insights into GBA influences on human decision-making through probiotics.

## Highlights

• Probiotics increased risk-taking vs. placebo in the Maastricht Gambling Task
• Response time increased, and choice optimality was not compromised
• Probiotics' effects were independent of TMS stimulation (VMPFC or SPL)
• VMPFC inhibition led to order-dependent (task repetition) increases in risk-taking

## Graphical abstract

**Figure:**
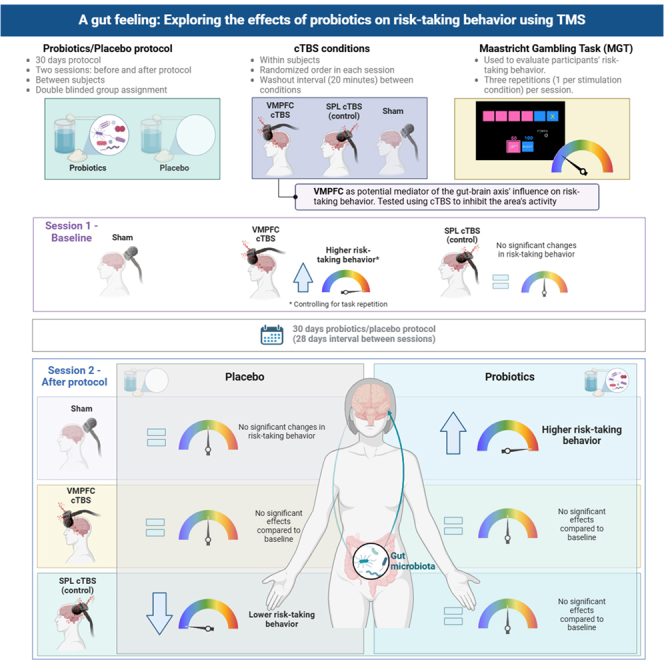

## Introduction

The human body typically hosts trillions of bacteria, viruses, and fungi, which are essential for homeostasis, health, and even brain development, forming a vital symbiotic relationship.^1^ The highest concentration of these bacteria is found in the gut, where they interact with the enteric nervous system (ENS) through a complex network of nerves. This interaction allows communication between the gut microbiota and the central nervous system (CNS), forming what is known as the microbiota-gut-brain axis (MGBA), or simply the gut-brain axis (GBA). The GBA has been shown to influence brain activity, potentially leading to changes in behavior.^2^^,^^3^ A well-known example of microbiome-influenced behavioral change is the infection of rodents with Toxoplasma gondii, which significantly reduces their fear of felines.^1^

Animal studies have highlighted the influence of gut microbiota on a wide range of behavioral responses. For instance, Sylvia and colleagues (2017) demonstrated that broad-spectrum antibiotics targeting the gut microbiota effectively reduced aggressive behavior in Siberian hamsters.^3^ Sudo and colleagues (2004) found that germ-free mice showed increased emotional reactivity, which was reversed following reconstitution with probiotics (composed of *Bifidobacterium infantis*).^4^ Bravo and colleagues (2011) showed that after a probiotics protocol (of *Lactobacillus rhamnosus*), mice exhibited reduced anxiety and depression-related behavior.^5^ An intriguing example comes from Tillmann and Wegener (2019), who showed that after prolonged intake of a multistrain probiotics composition (Ecologic Barrier, Winclove probiotics, The Netherlands), Flinders Sensitive Line (FSL) rats, an animal model of depression, displayed reduced risk-taking behavior^6^ (for a comprehensive review, please refer to Sylvia & Demas (2018)^7^).

These findings in animals provide a strong foundation for understanding similar GBA influences in humans. For instance, the modulation of GBA activity after prolonged intake of probiotics has been shown to reduce stress-related behavior and increase visuospatial memory performance (Bifidobacterium longum 1714),^8^ improve scores of anxiety and depression (*Lactobacillus helveticus* R0052 and *Bifidobacterium longum* R0175) in clinical,^9^ and healthy participants^10^ and reduce cognitive reactivity to sad mood (EcologicBarrier),^11^ among others. Another important cognitive process potentially impacted by the gut-brain axis is human decision-making.

Decision-making is a highly relevant topic, given its frequency and the profound individual and societal impact. Yet, only a few studies to date have explored the influence of the gut-brain axis on human decision-making. One line of evidence comes from food-related decision-making. Medawar et al. (2024) reported that 14 days of prebiotic supplementation (inulin) in overweight adults reduced brain activation in the ventral tegmental area and orbitofrontal cortex when exposed to hypercaloric stimuli compared to placebo. Both regions are central to reward valuation, suggesting that prebiotics attenuate reward-related neural responses.

Beyond food choices, Bagga and colleagues (2018), showed that probiotics altered emotional decision-making (Ecologic825 or OmniBiotic, Winclove probiotics, the Netherlands) and brain activity in regions including the parahippocampal gyrus, inferior thalamus, cingulum, and precuneus. These findings point to probiotic effects on brain networks linked to emotional processing, cognitive control, and decision-making.

Social decision-making was explored in the work of Falkensein and colleagues (2024), where participants received either a 7-week symbiotic protocol (multispecies Biotic Junior, MensSana, the Netherlands) or a placebo. Participants in the symbiotic group displayed a reduction in rejection rates of unfair options.^12^ Finally, Dantas and colleagues (2022) explored individual decision-making with healthy participants, focusing on intertemporal choices and risk-taking behavior. Their results show that participants who went through a 4-week probiotics protocol (EcologicBarrier) had reduced time discounting and a relative reduction in risk-taking behavior compared to those who received a placebo.^13^

Risk-taking behavior is an especially relevant type of decision-making, given its impact and frequent occurrence in daily life, from simple choices such as carrying an umbrella on a cloudy day for the risk of raining to multimillion transactions in the financial market dealing with financial risks. However, the relationship between GBA and human decision-making under risk is still not clearly understood. Studies investigating the relevance of the GBA in risk-taking behavior are, to date, only using animal models,^6^^,^^14^ which reinforces the importance of further investigation on the topic.

Another key question yet to be addressed is how exactly changes in one’s gut microbiota can lead to changes in risk-taking behavior in general. Although no studies have specifically examined changes in brain activity during risk-taking behavior influenced by probiotics, insights can be drawn from research on probiotics’ effects on brain activity and connectivity during the resting state. A consistent finding from these studies is the alteration of functional connectivity in three networks that overlap with brain areas involved in risk processing: the default mode network (DMN), salience network (SN), and frontoparietal network (FPN).^15^^,^^16^^,^^17^ A recent example is a study by Rode and colleagues (2022), which showed that prolonged probiotic intake significantly affects gray matter volume and functional connectivity between several brain areas. Of interest, their results show a significant increase in functional connectivity between the DMN and the medial prefrontal cortex (MPFC),^18^ an important area related to valuation and somatic integration in human decision-making and specifically in risk-taking behavior.^19^^,^^20^^,^^21^

While evidence of differential patterns of brain activation during tasks comparing probiotics to placebos is still scarce, a relevant example is the work by Tillisch and colleagues (2013). In this study, the authors explored differential brain activity after prolonged probiotics intake in female participants during an emotional attention task. Their results show a significant reduction in task-related response of a network that includes primary interoceptive and somatosensory, periaqueductal gray, prefrontal cortex, precuneus, basal ganglia, and the parahippocampal gyrus. Such a pattern of activation was associated with a potential dampening of emotional responsiveness due to the involvement of two specific prefrontal areas, the MPFC and the dorsolateral prefrontal cortex (DLPFC).^22^ Both the MPFC and DLPFC are known to play pivotal roles in decision-making under risk. Moreover, basal ganglia activity has been consistently correlated with value-base-decision-making.^23^

Based on these findings, we see the MPFC as a potential key area in the processing of signals from the GBA during decision-making. More specifically, the ventromedial prefrontal cortex (VMPFC) might mediate the observed changes in risk-taking behavior after the prolonged intake of probiotics. This area is thought to function as a hub for somatic information,^24^^,^^25^ integrating bodily signals into decision-making.^19^^,^^25^ The ENS constitutes an important source of these bodily signals, since the gut is the organ outside the CNS that presents the highest neural complexity.^26^ The VMPFC is also a key component of the network involved in processing decisions involving risk.^25^^,^^27^^,^^28^^,^^29^^,^^30^ Furthermore, the VMPFC, as part of the default mode network (DMN), is reported as one of the brain areas whose activity is affected by the prolonged intake of probiotics.^18^^,^^22^^,^^31^^,^^32^ Nevertheless, the role of the VMPFC in the interaction with probiotics during risk-taking behavior remains unclear.

We designed an exploratory study using transcranial magnetic stimulation (TMS) to clarify the potential role of the VMPFC in the GBA’s influence on risk-taking behavior. Using TMS, namely continuous theta-burst stimulation (cTBS), it is possible to temporarily inhibit the activity of the VMPFC. We could then test whether inhibiting the activity of this area also influences the effects of probiotics on risk-taking behavior, which would then confirm the pivotal role of the VMPFC as an important part of the network underlying the gut-brain axis’s influence on the processing of risk-taking behavior.

Although Dantas et al. (2022) advanced our understanding of the GBA’s influence on risk-taking behavior, their study did not provide definitive conclusions about the effects of probiotics. The placebo group showed an unexpected increase in risk-taking, attributed to a “house-money effect” or payoff-based belief distortion,^33^^,^^34^ where participants took more risks due to the money received between sessions as part of their compensation.^13^ This led to what appeared as a relative reduction in risk-taking in the probiotics group, making it unclear how probiotics would affect risk-taking if the placebo group’s behavior were stable. Similarly, studies using animal models have also reported abnormal or heightened baseline risk-taking (e.g., germ-free animals or depression-prone rats).^6^^,^^14^ These findings raise the question of how the GBA influences risk-taking in healthy humans when there are no behavioral distortions in the placebo group, meaning without baseline distortions. Although the literature on probiotics and human risk-taking behavior is still scarce, we formulated the following hypotheses: First, we expected that prolonged probiotics intake would lead to measurable changes in risk-taking behavior compared to placebo. Second, we hypothesized that if the ventromedial prefrontal cortex (VMPFC) plays a critical role in mediating these effects, then inhibiting its activity using cTBS would attenuate or reverse probiotics-related changes.

**Figure 1:**
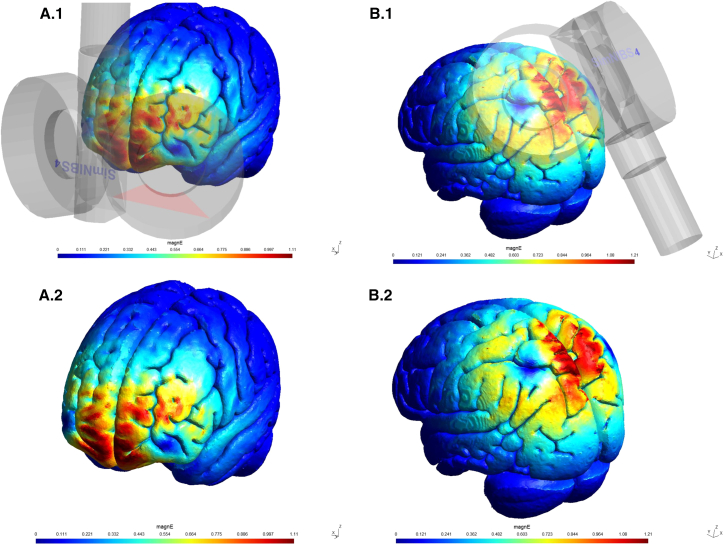
Electric field simulation for VMPFC and SPL stimulation A.1 and A.2 illustrate the electric field generated by the VMPFC stimulation over FpZ. A.1 also illustrates the coil positioning for this condition. B.1 and B.2 illustrate the electric field generated by the SPL stimulation over PZ. B.1 also illustrates coil positioning for SPL stimulation. Simulations were generated with SimNIBS 4 61 using Magventure Cool D-B80 coil model 62.

**Figure 2:**
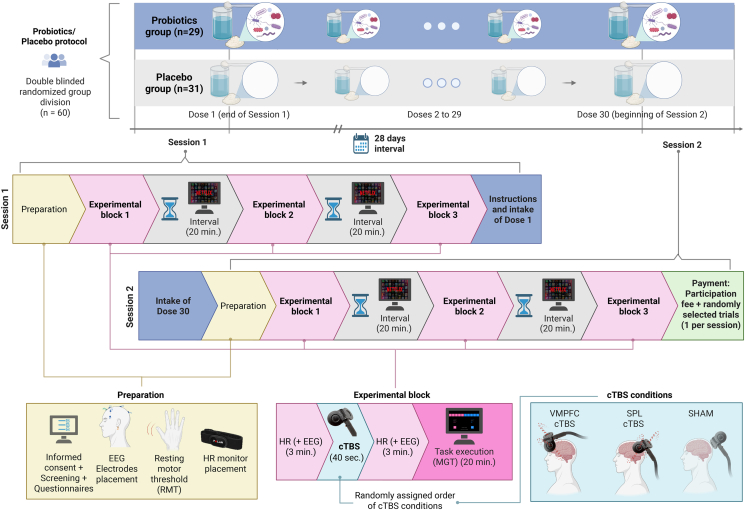
Experimental design Picture depicts the procedure of session 1 (above), before the 28 days probiotics/placebo protocol and session 2 (below) after the protocol. Both sessions included 3 blocks. During each block, participants went through two 3-minute EEG and HR measurements and received a different TMS treatment (VMPFC, SPL, or Sham) before executing the MGT. Blocks were separated by 20-minute washout periods. Each session included the 3 different TMS conditions in a randomized fashion.

To explore these hypotheses, we used a double-blind placebo-controlled experiment in which participants received probiotics (or a placebo) during an interval of 30 days. Participants’ risk-taking behavior was evaluated both before (session 1) and after the probiotics/placebo protocol (session 2) in a mixed design, using the MGT. The MGT is a computerized task that allows the measurement of our dependent variables, namely risk-taking behavior, choice optimality, and response time. In each session, participants received three rounds of cTBS. Sessions were divided into three experimental blocks, including heart rate (HR) measurements (and EEG, which is reported elsewhere), cTBS in one of the stimulation conditions (VMPFC, SPL, or sham), task execution, and a 20-min washout interval. Coil positioning and electric field simulation for each active condition can be seen in Figure 1. The study also included scales used to control for potential confounding factors. These scales were the Self-Assessment Manikin (SAM),^35^ the Brief Self-Control Scale (BSCS),^36^ a brief dietary questionnaire,^13^ and sections of the Global Preferences Survey (GPS)^37^ to evaluate participants’ time and risk preferences. Figure 2 illustrates the experimental design, and further details can be seen in the STAR Methods section.

**Figure 3:**
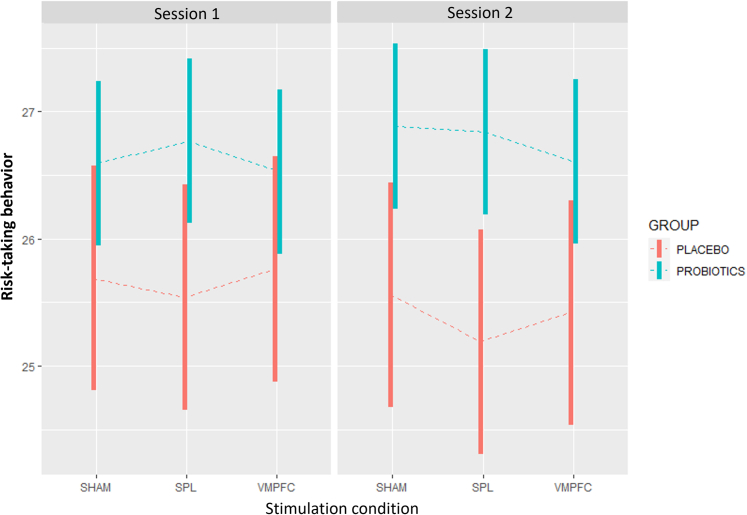
Average risk-taking behavior Figure depicts the average risk-taking behavior during session 1 (left box) and session 2 (right box) by group (probiotics in blue and placebo in red). Risk-taking behavior is displayed along the y-axis. Bars represent confidence intervals and dashed lines represent means. Different stimulation conditions are depicted in the y-axis (sham, SPL and VMPFC). Linear mixed models analyses were used for quantification. Analyses included 60 participants and 44375 observations.

**Figure 4:**
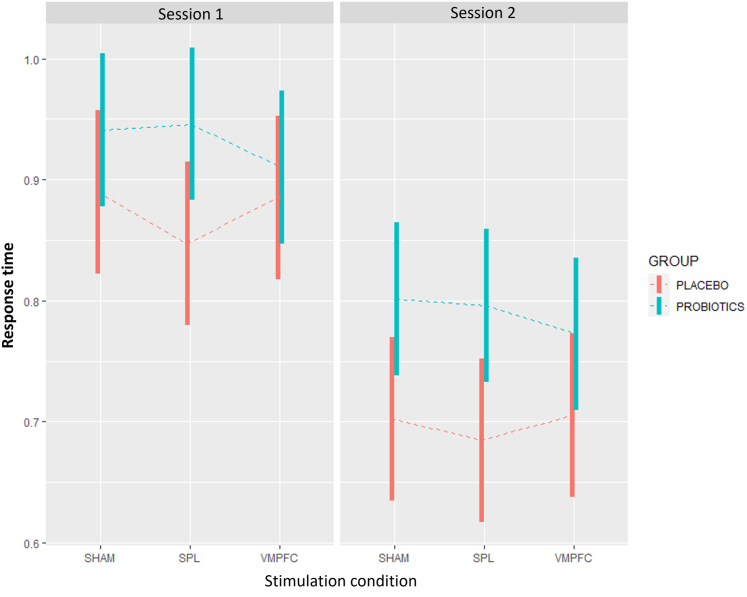
Average response time Figure depicts participants’ average response time during session 1 (left box) and session 2 (right box) by group (probiotics in blue and placebo in red). Response time is displayed along the y-axis. Bars represent confidence intervals and dashed lines represent means. Different stimulation conditions are depicted in the y-axis (sham, SPL and VMPFC). Linear mixed models analyses were used for quantification. Analyses included 60 participants and 43387 observations.

## Results

### Risk-taking behavior

We first examined participants’ risk-taking behavior across sessions, groups, and stimulation conditions. In the linear mixed model (LMM) without the repetition factor, the overall explanatory power was very high (conditional R^2^ = 0.92), reflecting that the model captured substantial variance. As expected, much of this variance was explained by trial-type differences and inter-individual variability rather than fixed factors (marginal R^2^ of 2.64e–03). The model intercept (placebo group, session 1, sham stimulation) was at 25.33 (95% CI [24.66, 25.99], t(44360) = 74.46, *p* < 0.001), indicating the average level of risk-taking at intercept (Table 1).

**Table 1:** Risk-taking behavior – LMM results

Predictors^a^	Estimates	std. Error	CI	*p*
**Risk-taking behavior (STD)**				

(Intercept)	25.33	0.34	24.66–25.99	**<0.001**
SESSION [2]	0	0.1	−0.20–0.19	0.973
GROUP [PROBIOTICS]	1.32	0.47	0.41–2.24	**0.004**
STIMULATION [SPL]	−0.03	0.1	−0.22–0.17	0.781
STIMULATION [VMPFC]	0.08	0.1	−0.11–0.27	0.405
SESSION [2] × GROUP [PROBIOTICS]	0.27	0.14	0.01–0.54	**0.043**
SESSION [2] × STIMULATION [SPL]	−0.28	0.14	−0.56–−0.01	0.042
SESSION [2] × STIMULATION [VMPFC]	−0.19	0.14	−0.47–0.08	0.161
GROUP [PROBIOTICS] × STIMULATION [SPL]	0.14	0.13	−0.12–0.41	0.282
SESSION [2] × GROUP [PROBIOTICS]	0.27	0.14	0.01–0.54	**0.043**
SESSION [2] × STIMULATION [SPL]	−0.28	0.14	−0.56–−0.01	**0.042**
SESSION [2] × STIMULATION [VMPFC]	−0.19	0.14	−0.47–0.08	0.161
GROUP [PROBIOTICS] × STIMULATION [SPL]	0.14	0.13	−0.12–0.41	0.282
GROUP [PROBIOTICS] × STIMULATION [VMPFC]	−0.15	0.13	−0.41–0.12	0.277
(SESSION [2] × GROUP [PROBIOTICS]) × STIMULATION [SPL]	0.13	0.19	−0.25–0.50	0.498
(SESSION [2] × GROUP [PROBIOTICS]) × STIMULATION [VMPFC]	−0.03	0.19	−0.40–0.35	0.883

**Random Effects**				

σ^2^	16.88			
τ_00__TRIALCODE:PARTICIPANT_	192.99			
τ_00__PARTICIPANT_	1.56			
ICC	0.92			
N _TRIALCODE_	125			
N _PARTICIPANT_	60			
Observations	44375			
Marginal R^2^/Conditional R^2^	0.003/0.920			
AIC	283138.646			

a Fixed effects from the primary LMM (formula: RISK ∼ SESSION + GROUP + STIMULATION + SESSION ∗ GROUP + SESSION ∗ STIMULATION + STIMULATION ∗ GROUP + SESSION ∗ GROUP ∗ STIMULATION). The model included TRIALCODE as random effects (formula: list(∼1 |TRIALCODE:PARTICIPANT, ∼1 | PARTICIPANT)).

Overall, there was no significant session effects, with no significant change in risk-taking from session 1 to session 2 across groups (*p* > 0.05). Contrast analyses confirmed that the placebo group maintained stable risk-taking levels over time (all *p* > 0.05; see Table 2).

**Table 2:** Risk-taking behavior – Contrast analyses

Risk-taking behavior (STD)^a^							
GROUP	STIM.	SESS.	contrast	Estim.	SE	z.ratio	p
PLACEBO	SHAM	.	SESSION2 - SESSION1	−0.003	0.098	−0.033	1
PROBIOTICS	SHAM	.	SESSION2 - SESSION1	0.272	0.094	2.896	0.054
PLACEBO	SPL	.	SESSION2 - SESSION1	−0.286	0.098	−2.915	0.051
PROBIOTICS	SPL	.	SESSION2 - SESSION1	0.118	0.093	1.275	0.931
PLACEBO	VMPFC	.	SESSION2 - SESSION1	−0.198	0.098	−2.015	0.444
PROBIOTICS	VMPFC	.	SESSION2 - SESSION1	0.0487	0.094	0.519	1
	SHAM	1	PROBIOTICS - PLACEBO	1.324	0.466	2.843	0.063
	SHAM	2	PROBIOTICS - PLACEBO	1.599	0.466	3.431	0.010
	SPL	1	PROBIOTICS - PLACEBO	1.469	0.466	3.154	0.024
	SPL	2	PROBIOTICS - PLACEBO	1.874	0.466	4.021	0.001
	VMPFC	1	PROBIOTICS - PLACEBO	1.178	0.466	2.529	0.150
	VMPFC	2	PROBIOTICS - PLACEBO	1.425	0.466	3.056	0.033
PLACEBO		1	SPL - SHAM	−0.027	0.098	−0.278	1
PLACEBO		1	VMPFC - SPL	0.109	0.098	1.11	0.973
PLACEBO		2	SPL - SHAM	−0.310	0.098	−3.16	0.024
PLACEBO		2	VMPFC - SPL	0.197	0.098	2.01	0.447
PROBIOTICS		1	SPL - SHAM	0.117	0.092	1.279	0.929
PROBIOTICS		1	VMPFC - SPL	−0.182	0.092	−1.982	0.469
PROBIOTICS		2	SPL - SHAM	−0.036	0.094	−0.379	1
PROBIOTICS		2	VMPFC - SPL	−0.252	0.094	−2.670	0.1034

a Fixed effects from the primary LMM (formula: RISK ∼ SESSION + GROUP + STIMULATION + SESSION ∗ GROUP + SESSION ∗ STIMULATION + STIMULATION ∗ GROUP + SESSION ∗ GROUP ∗ STIMULATION). The model included TRIALCODE as random effects (formula: list(∼1 |TRIALCODE:PARTICIPANT, ∼1 | PARTICIPANT)).

The probiotics effects, indicated by critical session × group interaction, indicated that probiotics significantly increased risk-taking compared to placebo (β = 0.27, 95% CI [8.70 × 10^−3^, 0.54], t(44 360) = 2.02, *p* = 0.043; standardized β = 0.02, 95% CI [5.98e-04, 0.04]). Planned contrasts confirmed this effect during sham stimulation in session 2 (estimate = 1.599, SE = 0.466, z = 3.431, *p* = 0.0097; Table 2). Thus, probiotics intake, rather than time or task repetition alone, accounted for the increase in risk-taking.

Although the groups were randomly assigned, we observed a modest baseline difference in session 1 when collapsing across stimulations, with the probiotics group showing slightly higher risk-taking than the placebo group (β = 1.32, 95% CI [0.41, 2.24], t(44 360) = 2.84, *p* = 0.004). To investigate this difference, we checked the effects of each stimulation condition.

Focusing on stimulation effects, follow-up contrasts localized that the initially observed differences between the probiotics and placebo groups in session 1 were due to the SPL condition only (estimate = 1.469, SE = 0.466, z = 3.154, *p* = 0.0238), while sham and VMPFC did not differ between groups (both *p* > 0.05; Table 2).

In session 1, SPL stimulation did not significantly alter risk-taking relative to sham (all *p* > 0.05; Table 1). In session 2, however, SPL stimulation reduced risk-taking in the placebo group (β = −0.28, 95% CI [−0.56, −0.01], t(44 360) = −2.04, *p* = 0.042), confirmed by contrasts (estimate = −0.310, SE = 0.098, z = −3.160, *p* = 0.0237; Table 2). VMPFC stimulation had no significant effects (*p* > 0.05).

To directly test whether the VMPFC mediated probiotics’ effects, we examined the interactions between the probiotics protocol and stimulation, focusing on the three-way session 2 x group probiotics × VMPFC stimulation interaction. Neither VMPFC nor SPL stimulation modulated the probiotics effect in session 2 (all *p* > 0.05; Table 1).

In sum, risk-taking behavior was stable in the placebo group but significantly increased in the probiotics group after the 4-week intake. Stimulation effects were limited to an SPL-related reduction in risk-taking within the placebo group at session 2. Crucially, probiotics-related increases in risk-taking were observed across stimulation conditions, indicating that the VMPFC does not mediate this effect (see full model estimates in Table 1 and contrasts in Table 2). These results are depicted in detail in Figure 3.

To account for potential task repetition effects (cf. Dantas et al., 2023), we ran a post hoc LMM on risk-taking behavior, controlling for task repetition, which means that the model included repetition (A, B, C) as an additional fixed factor. This model had a comparable overall fit (conditional R^2^ = 0.92) but explained slightly more variance with fixed effects (marginal R^2^ = 2.82e-03). The key findings were consistent with the primary analysis: probiotics intake increased risk-taking in session 2 (β = 0.59, 95% CI [0.09, 1.09], t(44 336) = 2.33, *p* = 0.020). Importantly, including repetition revealed additional effects of VMPFC stimulation, which increased risk-taking in session 1 (β = 0.52, 95% CI [0.17, 0.87], t(44 336) = 2.91, *p* = 0.004) but reduced risk-taking in session 2 (β = −0.69, 95% CI [−1.21, −0.17], t(44 336) = −2.61, *p* = 0.009). A significant interaction between VMPFC stimulation and repetition B (β = −1.18, *p* < 0.001) indicated that stimulation effects varied across task blocks. Finally, a session 2 x probiotics × SPL interaction appeared during repetition C (β = 1.29, 95% CI [0.18, 2.40], t(44 336) = 2.27, *p* = 0.023), suggesting a late-session modulation. Full model estimates and contrasts are reported in Tables S1 and S2 (supplemental information).

### Choice optimality

We next examined whether probiotics influenced the efficiency of decision-making. Choice optimality indicates if participants chose the highest expected value in each trial type (please refer to the section on Choice Optimality in Quantification and Statistical Analysis for further details). The LMM obtained showed substantial explanatory power (conditional R^2^ = 0.66), with most variance again explained by trial type and individual differences rather than fixed factors (marginal R^2^ of 3.33e-03). The intercept (placebo group, session 1, sham stimulation) was 0.85 (95% CI [0.83, 0.88], t(44 360) = 77.53, *p* < 0.001).

Choice optimality remained stable across sessions (*p* > 0.05), and the session × group interaction was not significant, indicating that prolonged probiotics intake did not affect participants’ ability to select options with higher expected value (Table S3, supplemental information).

There were no significant effects of stimulation, since neither VMPFC nor SPL stimulation significantly influenced choice optimality in either session (all *p* > 0.05), and there were no significant interactions with probiotics intake (Table S3).

Finally, we checked for potential baseline differences. Although a small group difference emerged in session 1 when collapsing across stimulation conditions (β = 0.03, 95% CI [4.33 × 10^−3^, 0.06], t(44 360) = 2.25, *p* = 0.025), follow-up contrasts confirmed no significant differences between groups after sham, VMPFC, or SPL stimulation (all *p* > 0.05; Table S4). These baseline differences were therefore considered noise.

### Response time

We next analyzed participants’ response times across sessions, groups, and stimulation conditions using an LMM. The model had substantial total explanatory power (conditional R^2^ = 0.43), with most variance attributable to trial type and individual differences rather than fixed effects (marginal R^2^ = 0.06). The intercept (placebo group, session 1, sham stimulation) was 0.89 s (95% CI [0.82, 0.96], t(43 372) = 25.86, *p* < 0.001) (Table 3).

**Table 3:** Response times – LMM results

Response times				
Predictors^a^	Estimates	std. Error	CI	*p*
(Intercept)	0.89	0.03	0.82–0.96	<0.001
SESSION [2]	−0.19	0.01	−0.20–−0.17	<0.001
GROUP [PROBIOTICS]	0.05	0.05	−0.04–0.14	0.279
STIMULATION [SPL]	−0.04	0.01	−0.06–−0.03	<0.001
STIMULATION [VMPFC]	0	0.01	−0.02–0.01	0.457
SESSION [2] × GROUP [PROBIOTICS]	0.05	0.01	0.03–0.07	<0.001
SESSION [2] × STIMULATION [SPL]	0.03	0.01	0.01–0.04	0.006
SESSION [2] × STIMULATION [VMPFC]	0.01	0.01	−0.01–0.03	0.412
GROUP [PROBIOTICS] × STIMULATION [SPL]	0.05	0.01	0.03–0.07	<0.001
GROUP [PROBIOTICS] × STIMULATION [VMPFC]	−0.03	0.01	−0.04–−0.01	0.004
(SESSION [2] × GROUP [PROBIOTICS]) × STIMULATION [SPL]	−0.04	0.01	−0.06–−0.01	0.006
(SESSION [2] × GROUP [PROBIOTICS]) × STIMULATION [VMPFC]	−0.01	0.01	−0.03–0.02	0.655

**Random Effects**				

σ^2^	0.07			
τ_00__TRIALCODE:PARTICIPANT_	0.02			
τ_00__PARTICIPANT_	0.03			
ICC	0.39			
N _TRIALCODE_	125			
N _PARTICIPANT_	60			
Observations	43387			
Marginal R^2^/Conditional R^2^	0.062/0.433			
AIC	15973.036			

a Fixed effects from the primary LMM (formula: ResponseTime ∼ SESSION + GROUP + CONDITION + SESSION ∗ GROUP + SESSION ∗ CONDITION + CONDITION ∗ GROUP + SESSION ∗ GROUP ∗ CONDITION). The model included TRIALCODE as random effects (formula: list(∼1 | TRIALCODE:PARTICIPANT, ∼1 | PARTICIPANT)).

A significant effect of session (comparing session 2 to session 1) was observed (beta = −0.19, 95% CI [-0.20, −0.17], t(43372) = −28.78, *p* < 0.001; Std. beta = −0.53, 95% CI [-0.56, −0.49]). Such results are confirmed by contrast analyses (Table 3, supplemental information). Overall, these results indicate that participants were faster in their responses during the second session.

The effects of probiotics, analyzed as the critical session × group interaction, indicated longer response times after probiotics intake (β = 0.05, 95% CI [0.03, 0.07], t(43 372) = 5.28, *p* < 0.001; standardized β = 0.13, 95% CI [0.08, 0.18]). However, this effect did not survive planned contrasts: response times after sham stimulation in session 2 did not differ significantly between groups (*p* > 0.05; Table 4).

**Table 4:** Response times – Contrast analyses

Response times^a^							
GROUP	STIM.	SESS.	contrast	Estim.	SE	z.ratio	p
PLACEBO	SHAM	.	SESSION2 - SESSION1	−0.187	0.006	−28.782	**<0.001**
PROBIOTICS	SHAM	.	SESSION2 - SESSION1	−0.140	0.006	−22.207	**<0.001**
PLACEBO	SPL	.	SESSION2 - SESSION1	−0.162	0.006	−24.927	**<0.001**
PROBIOTICS	SPL	.	SESSION2 - SESSION1	−0.150	0.006	−24.132	**<0.001**
PLACEBO	VMPFC	.	SESSION2 - SESSION1	−0.180	0.006	−27.545	**<0.001**
PROBIOTICS	VMPFC	.	SESSION2 - SESSION1	−0.138	0.006	−21.989	**<0.001**
	SHAM	1	PROBIOTICS - PLACEBO	0.051	0.047	1.083	0.976
	SHAM	2	PROBIOTICS - PLACEBO	0.099	0.047	2.097	0.378
	SPL	1	PROBIOTICS - PLACEBO	0.099	0.047	2.098	0.377
	SPL	2	PROBIOTICS - PLACEBO	0.111	0.047	2.363	0.218
	VMPFC	1	PROBIOTICS - PLACEBO	0.025	0.047	0.533	1
	VMPFC	2	PROBIOTICS - PLACEBO	0.067	0.047	1.427	0.862
PLACEBO		1	SPL - SHAM	−0.043	0.006	−6.573	**<0.001**
PLACEBO		1	VMPFC - SPL	0.038	0.006	5.814	**<0.001**
PLACEBO		2	SPL - SHAM	−0.018	0.006	−2.74	0.083
PLACEBO		2	VMPFC - SPL	0.020	0.006	3.155	**0.023**
PROBIOTICS		1	SPL - SHAM	0.005	0.006	0.786	0.998
PROBIOTICS		1	VMPFC - SPL	−0.036	0.006	−5.765	**<0.001**
PROBIOTICS		2	SPL - SHAM	−0.005	0.006	−0.841	0.997
PROBIOTICS		2	VMPFC - SPL	−0.024	0.006	−3.79	**0.002**

a Fixed effects from the primary LMM (formula: ResponseTime ∼ SESSION + GROUP + CONDITION + SESSION ∗ GROUP + SESSION ∗ CONDITION + CONDITION ∗ GROUP + SESSION ∗ GROUP ∗ CONDITION). The model included TRIALCODE as random effects (formula: list(∼1 | TRIALCODE:PARTICIPANT, ∼1 | PARTICIPANT)).

Regarding stimulation effects, in session 1, SPL stimulation reduced response times relative to sham (β = −0.04, 95% CI [−0.06, −0.03], t(43 372) = −6.57, *p* < 0.001). In session 2, by contrast, SPL stimulation increased response times (β = 0.03, 95% CI [7.15 × 10^−3^, 0.04], t(43 372) = 2.74, *p* = 0.006). VMPFC stimulation did not significantly affect response times in either session (all *p* > 0.05).

Afterward, we focused on the interactions between the group (probiotics or placebo) and stimulation. Despite the random allotment to groups, our results indicate significantly higher response times after SPL stimulation (beta = 0.05, 95% CI [0.03, 0.07], t(43372) = 5.31, *p* < 0.001; Std. beta = 0.13, 95% CI [0.08, 0.18]) and significantly lower response times after VMPFC stimulation (beta = −0.03, 95% CI [-0.04, −8.24e-03], t(43372) = −2.87, *p* = 0.004; Std. beta = −0.07, 95% CI [-0.12, −0.02]) in the probiotics group only, before the probiotics/placebo intake (session 1). To clarify such effects, we conducted contrast analyses (see Table 4, supplemental information). These results indicate a significant reduction in response time after SPL stimulation in the placebo group only, during session 1 (estimate = −0.043, SE = 0.006, z(43372) = −6.573, *p* < 0.001). Nevertheless, the two groups did not differ significantly before experimental manipulations (session 1 after sham stimulation) (*p* > 0.05).

To test a potential interaction between the probiotics and VMPFC stimulation, we investigated the interaction session 2 × probiotics × VMPFC stimulation, which yielded non-significant effects (*p* > 0.05). However, the interaction with the SPL stimulation led to a significant reduction in response time (beta = −0.04, 95% CI [-0.06, −0.01], t(43372) = −2.77, *p* = 0.006; Std. beta = −0.10, 95% CI [-0.17, −0.03]). These results potentially explain the overall effects of probiotics observed in the initial analysis, which collapsed results obtained after all three stimulation conditions (sham, SPL, and VMPFC). The effects of both probiotics and stimulation conditions on response time are shown in Figure 4 (full model estimates and contrasts are available in Tables 3 and 4).

We also explored HR changes across groups, sessions, and stimulation conditions. The LMM revealed a significant overall reduction in HR after stimulation compared to before stimulation, independent of condition (beta = −1.48, 95% CI [-2.47, −0.49], t(656) = −2.93, *p* = 0.003; Std. beta = −0.06, 95% CI [-0.11, −0.02]). No additional effects of session, group, or stimulation were observed (all *p* > 0.05). Thus, while HR decreased following stimulation in general, probiotics intake did not influence cardiac activity. Full results are provided in Table S5 (supplemental information).

Finally, we analyzed potential changes in control measures to exclude confounding influences. No significant group differences were observed at baseline (session 1). There were no changes across sessions in self-control (BSCS), mood, and arousal (SAM), dietary habits, or risk/time preferences (GPS) (all *p* > 0.05). Importantly, the probiotics × session interaction was not significant for any of the scales (all *p* > 0.05). These findings confirm that the behavioral effects reported above were not driven by changes in mood, diet, or self-control. Full model estimates for all scales are available in Tables S6–S13 (supplemental information).

## Discussion

This study investigated whether the modulation of the gut-brain axis influences human decision-making, with a focus on risk-taking behavior. Our methodology combined a four-week double-blinded protocol of either probiotics or placebo with TMS targeting the VMPFC, using the SPL and sham as controls. These specific techniques were used to investigate the potential role of the VMPFC in the interaction with probiotics during risk-taking behavior.

Our main finding is that prolonged probiotics intake significantly increased risk-taking behavior compared to placebo. Participants in the placebo group maintained stable levels of risk-taking across sessions, whereas those in the probiotics group chose significantly riskier options in the computerized gambling task (MGT). This effect was observed in the context of a stable baseline, which strengthens the interpretation that it was driven by probiotics intake rather than task repetition or time effects. These findings contrast with Dantas et al. (2022), who reported a relative reduction in risk-taking following probiotics intake. A likely explanation is that their placebo group showed atypical baseline increases in risk-taking, possibly due to a house-money effect or payoff-based belief distortion, potentially triggered by participant compensation during an intertemporal choice task.^13^^,^^33^^,^^34^ In our study, careful design choices minimized such distortions: the placebo group remained stable across sessions, the task was restricted to risk-taking only, and potential confounds such as mood, diet, and self-control were monitored.

Studies examining the effects of probiotics on risk-taking behavior in both human and animal models often rely on distorted baselines. This includes Dantas and colleagues (2022), as well as previous studies. These studies used either germ-free animals or the Flinders Sensitive Line rat model of depression as baselines, both displaying abnormal risk-taking behavior.^6^^,^^14^ In all these examples, the baseline shows abnormal risk-taking behavior, which was “normalized” after prolonged probiotics intake.

Importantly, our results show a significant yet modest difference in risk-taking behavior between groups during session 1, which indicates differences between groups before the probiotics/placebo protocol. This difference could indicate sampling bias with pre-existing differences between groups despite the double-blinded procedure. These results can be attributed to the significant differences between groups observed after SPL stimulation and during session 1 (significant interaction between SPL stimulation and group probiotics before the probiotics intake). However, the SPL stimulation alone did not yield significant effects on risk-taking behavior. Furthermore, there were no significant differences after VMPFC and (more importantly) sham stimulation. No significant differences between groups after sham stimulation confirmed that there were no significant differences between groups when no active experimental manipulation was in place. Considering the double blinded placebo-controlled probiotics manipulation, and the non-significant effects of SPL stimulation alone, such differences between groups before the probiotics/placebo protocol (session 1) are considered unexpected but should nevertheless be noted.

Overall, our study provides a key contribution to this literature by investigating the effects of probiotics on risk-taking in a non-distorted baseline context, with two important implications. First, the impact of probiotics on risk-taking behavior appears to depend on baseline risk-taking patterns. When baseline risk-taking behavior is stable, meaning that there are no distortions in risk-taking behavior in the placebo group over time, probiotics lead to an increase in risk-taking. In contrast, when the baseline behavior was atypical, for instance, with abnormal increases in risk-taking behavior in the placebo group, probiotics seemed to stabilize participants’ risk-taking. Second, the study demonstrates that manipulating gut microbiota can influence higher-order decision-making under risk, a critical insight given the current scarcity of research on this topic.

To further our insights into the mechanism via which the GBA influences human decision-making, we also examined choice optimality. One can question whether higher risk-taking reflects a detrimental effect of probiotics intake on decision quality. Therefore, we explored the potential effects of this manipulation on choice optimality, which indicated whether participants chose the option with a higher expected value in each trial presented. Hence, choice optimality can be taken as an indicator of participants’ choice efficiency or rationality.^38^^,^^39^

Our results show that choice optimality is a rather stable measure, as participants consistently choose options with a higher expected value with very little fluctuation. Although there is an apparent difference in choice optimality between groups (probiotics vs. placebo) during the first session (before the probiotics/placebo protocol), this difference does not survive contrast analyses, being driven by the effects of the VMPFC stimulation during session 1 in this specific group only and therefore is not statistically significant. Despite the double-blinded procedure, slight differences between groups at session 1 were observed. The source of such baseline differences can only be speculated at this point and did not affect further analyses.

Looking specifically at the results of interest, particularly the effects of the probiotics protocol on choice optimality, our findings showed no significant differences. These null results provide valuable information in our effort to better comprehend how the gut-brain axis influences human decision-making. Taking together the increase in risk-taking behavior and the stable level of choice optimality observed after the probiotics manipulation, we can conclude that the increase in risk-taking behavior cannot be attributed to a reduction in participants’ ability to make optimal decisions. Therefore, our results indicate that participants opted for higher risks without compromising their overall choice optimality.

Another potential explanation for the effect of changes in the GBA due to the prolonged intake of probiotics on risk-taking behavior could be attributed to an increase in automatic responses.^40^ To explore this possibility, we analyzed participants’ response time. Our results show that participants’ responses were significantly faster during the second experimental session, regardless of group (probiotics or placebo) or stimulation condition. Such effects were likely due to practice with the task and habituation to the experimental setting, which did not impact the participants’ risk-taking behavior. However, participants in the probiotics group exhibited a significant increase in response times compared to the placebo group. This increase in response time indicates that the higher levels of risk-taking behavior observed after the probiotics protocol cannot be attributed to more automatic and therefore faster responses. In fact, the observed results indicated that the increase in risk-taking behavior was deliberate.

Nevertheless, it is important to point out that the effects of probiotics on response time did not yield significant results in the contrast analyses. This means that the results obtained by the probiotics group during sham stimulation were not significantly different from those obtained by the placebo group, even though the overall effects of the probiotics manipulation were significant when including all stimulation conditions during session 2. The differences were only significant when taking into account the response times of participants in the probiotics group in all stimulation conditions, while the effects of interactions between probiotics and each stimulation condition (SPL or VMPFC) were not significant. Therefore, the effects of the probiotics manipulation on response time should be taken with caution. However, it is reasonable to conclude that the higher risk-taking behavior observed after probiotics intake cannot be attributed to faster and more automatic responses.

We hypothesized that probiotics’ effects on risk-taking behavior would be mediated by the VMPFC. The VMPFC was chosen since it is a main component of the DMN, which, according to a number of studies, has its activity affected by the prolonged intake of probiotics.^16^^,^^18^^,^^31^ This area has also been hypothesized as a hub, integrating somatic information in human decision-making.^19^^,^^24^ The mediation role of the VMPFC was therefore tested using cTBS to create a temporary deactivation of this area. The SPL was used as a control position due to its anatomical distance from the primary target and lack of involvement in the DMN.

In line with prior findings (e.g., Dantas et al., 2023), VMPFC inhibition in session 1 led to increased risk-taking behavior, consistent with neuropsychological studies showing that patients with focal VMPFC lesions display elevated risk-taking.^25^^,^^41^ However, the effects of this stimulation protocol were only significant when controlling for task repetition. According to our results, while there were significant increases in risk-taking behavior after VMPFC cTBS during the first and third task repetitions, the stimulation did not produce significant effects during the second repetition of the task. It is noteworthy that participants repeated the same task 3 times during each session, and the order of stimulation conditions was randomized. The reasons for the variable effects of VMPFC inhibition on risk-taking behavior can only be speculated. One potential explanation could be differences in cortical excitability.^42^ Although our design aimed at maintaining stable conditions throughout the session, it is possible that participants’ alertness may have fluctuated during the sessions, which could have affected the effectiveness of cTBS.

Interestingly, during session 2, the same stimulation protocol (cTBS over the VMPFC) led to effects in the opposite direction, meaning a reduction in risk-taking behavior in both probiotics and placebo groups. These findings diverge from lesion studies and suggest that transient cTBS-induced modulation may not map directly onto the effects of permanent focal damage. Possible explanations include dynamic changes in cortical excitability between sessions, which are potentially influenced by task familiarity or adaptation to the experimental context. Given the limited number of studies examining behavioral consequences of VMPFC inhibition with cTBS, further research is needed to clarify under which conditions cTBS effects align with, or deviate from, lesion-based evidence.

Regardless, the method in our study aimed to investigate the VMPFC’s role in GBA interaction during risk-taking behavior. To this end, it is sufficient to assume that the stimulation protocol effectively inhibits VMPFC activity, as indicated by significant behavioral effects. Therefore, the analysis of the interaction between VMPFC inhibition and the probiotics protocol is of the most interest for our study. Nevertheless, contrary to our initial hypothesis, VMPFC deactivation did not significantly interact with the effectiveness of probiotics manipulation. Our results suggest that the probiotics' effects could not be modulated with VMPFC inhibition using cTBS. These results indicate that although the VMPFC appears as a common denominator in many neuroimaging studies exploring changes in resting-state brain activity after prolonged intake of probiotics, this area does not seem to play a pivotal role specifically in the GBA’s communication during human risk-taking behavior, since no significant interactions between the VMPFC inhibition and the probiotics protocol were observed. Although informative, the results of the stimulation conditions do not help clarify the mechanisms via which the gut-brain axis affects human risk-taking behavior.

Regarding HR measurements and control scales, we found no significant differences in average HR between probiotics and placebo groups. This null result should be interpreted cautiously, as our analyses are based on resting-state measurements using a commercial HR monitor that, although used in several studies, does not supply highly detailed data.^43^ Hence, it is possible that subtle physiological effects were not detected due to the limited sensitivity of the commercial HR monitor used in this study. More sensitive approaches, such as electrocardiography (ECG) with the subsequent analysis of heart rate variability (HRV), could reveal finer-grained GBA-related effects on cardiac activity. Future studies should therefore incorporate these methods to strengthen the physiological evidence for probiotics’ impact on brain-body interactions.

Similarly, no significant changes were observed in any of the control scales assessing self-control, mood, arousal, risk, or time preferences. This confirms that confounding factors did not change across sessions and strengthens the interpretation that the observed increase in risk-taking was driven by probiotics intake rather than external influences. The placebo group in particular maintained stable behavior across sessions, providing a reliable baseline against which to interpret the probiotics effect.

Our findings underscore the relevance of the gut-brain axis in shaping human risk-taking behavior. Specifically, we establish a direct effect of probiotics intake, as participants showed a significant increase in risky choices after prolonged probiotics intake compared to placebo in the context of a stable baseline. This increase in risk-taking behavior was accompanied by consistent choice optimality and longer response times, suggesting that the changes in behavior do not reflect detrimental or impulsive responding, but rather a shift in decision strategy. A plausible broader mechanistic hypothesis is that probiotics modulate emotional regulation and/or reward valuation processes, thereby biasing decision-making under risk. Together, these findings contribute to a more precise understanding of how the gut-brain axis influences human cognition and behavior.

Another potential explanation for the increased risk-taking behavior is that probiotics intake would lead to better information integration. Previous studies have indicated that probiotics supplementation could lead to higher cognitive performance.^44^ This better integration would, in turn, reduce risk aversion.^45^ Better information integration could also lead to more efficient mental calculations and a consequent increase in choice optimality. Nevertheless, we did not see improvements in choice optimality, which confirms this explanation. Although plausible, this hypothesis would require further testing, including additional behavioral measures.

It is also possible that probiotics intake improves emotional regulation. A number of studies indicate that probiotics supplementation reduces reactivity to stress and emotional reactivity to sad mood.^11^^,^^44^^,^^46^ Risk and uncertainty are known to generate emotional cues that ultimately lead to risk aversion.^47^^,^^48^ Therefore, it is possible that a prolonged intake of probiotics would lead to higher emotional regulation and, consequently, to a higher tolerability of risk. Again, this hypothesis requires further exploration.

Finally, another potential explanation for the increases in risk-taking behavior is that probiotics supplementation would modulate participants’ perception of reward. Growing evidence demonstrates important interactions between the GBA and the brain’s reward network, which goes beyond food reward, as initially expected.^49^^,^^50^^,^^51^ Although research with healthy humans in this direction is still lacking, gut microbiota imbalance has been associated with substance abuse disorders, such as alcohol, psychostimulants, and opiates.^51^ In the context of risk-taking behavior, we can hypothesize that the gut microbiota would modulate one’s risk-taking by affecting the attractiveness of external reward. This could explain the reduction in risk-taking seen when there is an abnormal high baseline and an increase in risk-taking in healthy participants with a normal baseline, as observed in our results. Yet, further investigation is needed.

Although we successfully modulated risk-taking behavior targeting the VMPFC using cTBS (when controlling for task repetition), this stimulation condition did not significantly interact with the effects of probiotics, suggesting that the VMPFC is not crucial in integrating GBA signals during the processing of human decision-making under risk. Our study adds to the existing literature by reinforcing the importance of the GBA in the processing of risk-taking behavior and ruling out the VMPFC as a key brain region mediating the impact of probiotics on decision-making under risk.

Nevertheless, evidence suggests that other cortical and subcortical regions, rather than the VMPFC, may be critical for mediating the effects of probiotics on decision-making. Probiotics intake has been reported to alter activity in networks such as the DMN, SN, and FPN.^15^^,^^16^^,^^17^ These networks are strongly implicated in cognitive processes such as valuation, working memory, and cognitive control, all of which are central to risk processing.^23^^,^^52^^,^^53^ Future studies should therefore replicate our experimental design while targeting additional cortical areas that compose these networks, with the use of cTBS. The use of neuroimaging methods (such as fMRI) and connectivity analyses for target definition is advised. With cTBS, it is possible to clarify which of these areas might be pivotal for the mediation of signals coming from the ENS during human decision-making. Considering the methodological limitations of this method, only cortical areas can be targeted using cTBS. Based on the systematic review of Mulder and colleagues (2023), a few potential new targets can be highlighted, such as the angular gyrus (DMN), the middle temporal gyrus (DMN and FPN), and the DLPFC (FPN).^17^ Another potential target mentioned in this review is the precuneus (DMN). However, considering our use of a deeper reach coil to apply cTBS over the SPL as a control, we can consider that this area was already stimulated in our study. And yet our results indicate no significant interactions between SPL stimulation and the probiotics protocol, also excluding this area as a potential mediator of the observed effects of probiotics intake in risk-taking behavior.

Although unrelated to our main hypotheses, it is noteworthy that our results show effects of applying cTBS over the SPL on participants’ response times. SPL inhibition led to significant reductions in response times during the first session and a significant increase in response times during the second session. This suggests that the effects of SPL stimulation may be mediated by the participant’s habituation, which means that SPL stimulation would lead to reductions in response time when there is little or no habituation to the task and increases in response time if a higher level of habituation is present. One potential explanation would be the role of the SPL in information integration, which is crucial for learning processes and subsequent habituation.^54^ However, the SPL also plays a significant role in other cognitive processes, such as working memory,^55^ episodic memory,^56^ and attention.^57^ Therefore, future studies should investigate the specific cognitive processes affected by this stimulation protocol and its effects in different directions observed in each session.

In conclusion, this work highlights the significance of the GBA in human decision-making under risk by demonstrating that prolonged probiotics intake is associated with increases in risk-taking behavior. Our findings reveal that while probiotics intake does not affect choice optimality, it is linked to longer response times compared to placebo. These findings contribute to our understanding of how probiotics intake influences risk-taking behavior in humans. Additionally, we observed behavioral modulation through cTBS inhibiting the VMPFC using cTBS when controlling for task repetition effects. However, the interaction between VMPFC inhibition and the probiotics protocol did not yield significant results, suggesting that, in the context of our experiment, this area does not seem to play a pivotal role in the mediation of signals from the GBA during the processing of risk-taking behavior. Nonetheless, further research is necessary to fully comprehend the underlying mechanisms through which the GBA affects human decision-making under risk. Understanding the effects of probiotics on risk-taking behavior could have important implications for developing complete models that predict individual and collective risk-taking behavior. Additionally, these findings may inform the development of potential clinical interventions for patients with atypical patterns of risk-taking behavior.

### Limitations of the study

The present work explores the influence of the GBA in human risk-taking behavior, testing the potential role of the VMPFC in this process. To investigate the GBA’s influence on human risk-taking behavior, we use a 4-week probiotics protocol based on the effectiveness of such a procedure in previous studies.^31^^,^^58^^,^^59^ However, a limitation of the present study is the absence of stool sample analyses to verify microbiome changes induced by the probiotic intervention. Gut microbiome analyses using, for example, 16s rRNA gene amplicon sequencing (such as in Aarts et al., 2017) or shotgun metagenomic sequencing (such as in Kelsey et al., 2021) can provide an important manipulation check and relevant insights to better understand the potential role of specific bacterial strains in the observed behavioral effects.^49^^,^^60^ Compositional analyses and diversity analyses (such as alpha and beta diversity) can also help clarify what type of microbiota changes are driving changes in human cognition.^17^ Future studies should therefore include such analyses to validate and refine interpretations of gut-brain axis effects on human decision-making.

A further limitation is the use of a multistrain probiotics combination (EcologicBarrier), which prevents the identification of strain-specific contributions. It remains possible that the observed behavioral results are due to specific strains in EcologicBarrier or to the combination of multiple of these strains. Future studies should therefore employ single-strain and comparative designs to determine which strains are most relevant for modulating decision-making under risk.

Our study also hypothesized that the VMPFC plays a pivotal role in the GBA’s communication during risk-taking behavior. However, this assumption derives from studies exploring the GBA’s influence on brain activity during the resting state. Therefore, it is plausible that different brain areas and networks would be involved in the processing of the GBA’s signals during decision-making. However, this investigation requires further investigation using neuroimaging during the task. The results of this investigation could indicate targets for future studies using TMS to modulate the GBA’s effects on human risk-taking behavior and help clarify how information from the gut is processed in the CNS during decision-making.

## Resource availability

### Lead contact

Requests for further information and resources should be directed to and will be fulfilled by the lead contact, Aline M. Dantas (a.dantas@maastrichtuniversity.nl).

### Materials availability

This study did not generate new unique reagents.

### Data and code availability

• Data: De-identified human behavioral and heart rate data have been deposited at Mendeley Data as doi: https://doi.org/10.17632/dnphv8wjc5.2. They are publicly available as of the date of publication.
• Code: All original code has been deposited at Mendeley Data and is publicly available at doi: https://doi.org/10.17632/dnphv8wjc5.2. as of the date of publication.
• Additional information: Any additional information required to reanalyze the data reported in this article is available from the lead contact upon request.

## Acknowledgments

We would like to acknowledge the non-author contribution of the students who supported the data collection in this study. We also acknowledge the support received by the Limburg University Fund/SWOL, the Graduate School of Business and Economics (GSBE), the School of Business and Economics, and the Faculty of Psychology and Neurosciences at 10.13039/501100001835Maastricht University. Finally, we acknowledge the support provided by Winclove Probiotics B.V., who supplied our probiotics and placebo while allowing us to have absolute scientific independence to report the results with absolute integrity.

## Author contributions

Methodology: A.M.D., T.S., E.B., and P.J. Investigation: A.M.D. Visualization: A.M.D. Supervision: T.S., E.B., and P.J. Writing – original draft: A.M.D. Writing – review and editing: A.M.D., T.S., E.B., and P.J.

## Declaration of interests

The authors declare no competing interests.

## Declaration of generative AI and AI-assisted technologies in the writing process

During the preparation of this work, the author(s) used the Scribendi AI tool exclusively for grammar and style corrections, with no involvement in content generation. After using this tool or service, the author(s) reviewed and edited the content as needed and take(s) full responsibility for the content of the publication.

## STAR★Methods

### Key resources table

**Table:** 

REAGENT or RESOURCE	SOURCE	IDENTIFIER
**Bacterial and virus strains**		

*Bifidobacterium bifidum* W23	Winclove Probiotics	Ecologic® Barrier
*Bifidobacterium lactis* W51	Winclove Probiotics	Ecologic® Barrier
*Bifidobacterium lactis* W52	Winclove Probiotics	Ecologic® Barrier
*Lactobacillus acidophilus* W37	Winclove Probiotics	Ecologic® Barrier
*Lactobacillus brevis* W63	Winclove Probiotics	Ecologic® Barrier
*Lactobacillus casei* W56	Winclove Probiotics	Ecologic® Barrier
*Lactobacillus salivarius* W24	Winclove Probiotics	Ecologic® Barrier
*Lactococcus lactis* W19	Winclove Probiotics	Ecologic® Barrier
*Lactococcus lactis* W58	Winclove Probiotics	Ecologic® Barrier

**Software and algorithms**		

Biorender	BioRender.com	https://BioRender.com
Matlab	MathWorks	https://nl.mathworks.com/products/matlab.html
R Studio	Posit	https://posit.co/
R	r-project	https://www.r-project.org/
SimNIBS	SimNIBS	https://simnibs.github.io/simnibs/build/html/index.html

**Deposited data**		

	Mendeley data	Doi: https://doi.org/10.17632/dnphv8wjc5.2

**Other**		

MagVenture ×100 stimulator	Magventure	https://magventure.com/
Polar H10 chest strap	Polar	https://www.polar.com/

### Experimental model and study participant details

#### Overview of research design

In this study, we used a mixed design, including a between-subjects, 4-week double-blinded, and placebo-controlled probiotics protocol, combined with the administration of three cTBS conditions (VMPFC, SPL, and sham) in a single blinded within-subjects fashion. Each participant received a kit containing 30 sachets of either probiotics or placebo. All kits were prepared by the probiotics/placebo supplier (Winclove), assigning participants to either probiotics or placebo, in a randomized fashion. The information on the content of individual kits, which was identified by participant numbers in separate sealed envelopes, was kept blinded until the end of the experiment. This procedure allowed keeping the group assignment double-blinded. The study included two sessions, each divided into three experimental blocks. During each block, participants had their HR measured (and EEG, which is reported elsewhere), received TMS (in one of the experimental conditions), and then performed a complete run of the experimental task, the Maastricht Gambling Task (MGT). The two sessions were separated by a 28-day interval (+/- 1). During this interval, participants were instructed to take daily doses of placebo/probiotics. EEG measurements were also performed during this study. Nevertheless, due to the independence of EEG measurements from stimulation and task, the complexity of these findings, and their exploratory character, the EEG results are considered beyond the scope of this paper. Participants’ risk-taking behavior was analyzed both across groups (probiotics vs. placebo) and within groups across stimulation conditions.

#### Study participant details

*A priori* power analysis was conducted based on the behavioral effect size reported by Messaoudi et al. (2011), who tested the same probiotic formulation in healthy participants (partial η^2^ = 0.691, equivalent to f = 1.4954). Assuming one between-subject factor (group: probiotic vs. placebo) and two within-subject factors (session [2 levels] × stimulation [3 levels]), the required sample size was estimated at 16 participants per group. To ensure sufficient power and comparability with related studies [15,61,62], we recruited 30 participants per group (N = 60).

67 participants were recruited using posters on campus and social media, targeting the local academic community. Three participants dropped out due to discomfort during the stimulation, one participant dropped out due to a COVID-19 infection, and three participants were excluded due to nonconformity with the probiotics protocol according to self-report and hence did not take part in session 2. Therefore, 60 healthy, right-handed adult participants (36 women) with normal or corrected-to-normal vision and an average age of 22.8 years (SD = 4.57) concluded the experiment (29 in the probiotics group). All participants gave written informed consent after being introduced to the experiment and screened for safety for the use of probiotics and TMS (Dantas et al., 2022; Rossi et al., 2009). The study was approved by the Ethics Review Committee Psychology and Neuroscience (ERCPN) of Maastricht University, the Netherlands (OZL_208_15_05_2019) and carried out in accordance with the standards set by the Declaration of Helsinki (Fortaleza Amendments).

During the 30 days of taking placebo/probiotics, participants were asked not to consume more than two units of alcohol or any drugs, including antibiotics or other probiotics. Participants were also requested to maintain a stable diet during the protocol. Dietary patterns and potential changes were evaluated using questionnaires before and after the completion of the protocol. Participants’ compensation was based on their outcome in the risk-taking task in the form of vouchers with monetary value in local commerce, paid at the completion of participation (end of session 2).

### Method details

#### Procedures

Participants were invited to our laboratory at Maastricht University, where they were properly informed about the study procedure, experimental methods, and potential risks. Subsequently, participants were screened for the safe use of probiotics and TMS. Informed consent was collected prior to the start of the experiment’s start. The study included two sessions, separated by an interval of 28 days.

Each session started with a series of scales (SAM, BSCS, brief dietary questionnaire, and sections of the GPS). conducted via Qualtrics, replicating Dantas and colleagues (2022). Please see the following section, Scales, for further details. Afterwards, the HR monitor and EEG cap were set up. TMS stimulation targets were marked following the international 10-20 EEG system, over FpZ (VMPFC) and SPL (Pz). The positioning for sham TMS was alternated between FpZ and Pz in a randomized fashion. Only in the first session was the resting motor threshold (RMT) of participants determined. RTM was defined as the lowest stimulation intensity needed to elicit a visible contraction of the left abductor pollicis brevis (APB) in 5 out of 10 pulses after stimulating the right motor cortex.

At the beginning of the session, participants were instructed on the MGT and completed 10 practice trials before the MGT’s execution. Each session included three experimental blocks, each composed of an EEG and HR measurement (both for 3 minutes, eyes closed), 40 seconds of stimulation (which was either over the VMPFC, SPL, or sham in randomized order), and a second EEG and HR measurement immediately after the stimulation (3 minutes, eyes closed), followed by task execution. Each block included the completion of 250 valid trials of the MGT, which took around 20 minutes. Experimental blocks were separated by a washout period of 20 minutes, during which participants watched a neutral television show. Participants were free to choose a TV show from a streaming platform with the restriction that it could not be extremely exciting or arousing. Participants’ behavior was monitored by the researchers during the interval, guaranteeing constant length and proper choice of TV show according to the established restrictions.

After completion of the three blocks, a random trial was selected for payment. To this end, participants used an online random number generator to select two numbers indicating the block (between 1 and 3) and trial (between 11 and 261, excluding the first 10 practice trials). The outcome of the selected block and trial was added to the participant’s final payoff to be paid at the end of her participation (session 2).

At the end of session 1, participants received their first dose of probiotics/placebo and a kit containing the remaining 29 doses in individual sachets. Participants were instructed to take a daily dose for the next 28 days, during which they received daily email reminders. Participants were also instructed to report any missing doses and bring the kit containing any remaining sachets to the second session with the aim of increasing compliance. Kits were identical in presentation.

The second session took place on the 30th (+/- 1) day of the experimental period, following the structure previously described, with the exception that session 2 was preceded by participants taking the last dose of probiotics/placebo, a pre-experimental check, and was concluded with participants’ payment. The pre-experimental check was then conducted to guarantee that the responses reported in their screening forms remained valid. Moreover, in this step, they were asked to report any missed doses of probiotics/placebo between sessions. Participants who missed three or more doses were excluded from the sample. Participants’ payment included an hourly participation compensation and the MGT payoff obtained in the two sessions. Unblinding of group division (between placebo and probiotics) was done by the conclusion of data collection.

#### Maastricht gambling task (MGT)

The MGT is a computerized task developed by Dantas et al. (2021) to elicit and evaluate risk-taking behavior. The task presents clear probabilities in each trial by showing six colored boxes (pink or blue), according to the classical economic definition of risk (Weber & Johnson, 2009). Participants were asked to guess the color of the box that hides a token (yellow X), which was randomly hidden behind one of the boxes in each trial. The color distribution of boxes is random and can range from 1 to 5 pink boxes, with the remaining boxes blue. Each color is associated with a potential reward, randomly picked from 5, 25, 50, 75, or 100 points. If participants guess correctly (hit), they earn the associated number of points in that trial. Otherwise (miss), they earn zero points. All trials were independent, meaning that participants started anew at each trial. All possible color combinations of boxes and associated rewards are presented twice, with 125 unique trials and 250 trials in total. The task was developed based on the widely used “risk task”,^61^ also known as the Cambridge Gambling Task (CGT). However, this protocol controls for memory effects with independent trials repeated in a randomized order and for loss aversion, since participants cannot lose points but only earn zero points in the case of a miss. By the end of each session, a random trial is selected for payment, with 1 point equivalent to € 0,10. Please see Dantas et al. (2021) for a detailed description of the task. The MGT allows for the calculation of multiple dependent variables of interest, and we focus on participants’ risk-taking behavior, response times, and choice of the options with the highest expected value, which we define as choice optimality. Note that we call this choice optimality because choosing the higher expected value in our task is optimal for risk-neutral participants, but not for all risk attitudes.

#### Probiotics

For probiotics/placebo manipulation, we used the probiotics Ecologic®Barrier (Ecologic®Barrier, Winclove Probiotics, The Netherlands), previously used successfully in similar studies.^6^^,^^13^^,^^62^ Ecologic®Barrier is composed of Bifidobacterium bifidum W23, Bifidobacterium lactis W52, Lactobacillus acidophilus W37, Lactobacillus brevis W63, L. casei W56, Lactobacillus salivarius W24, and Lactococcus lactis (W19 and W58), distributed as sachets containing 2 g of freeze-dried powder of the PF for oral intake (mixed with water). The protocol for both probiotics and placebo groups included intake of 30 doses for 30 days, with the placebo group receiving a bacteria-free placebo created by the same laboratory instead. The placebo sachets were based on corn starch and identical to the probiotics composition, both visually and in flavor.

#### TMS

During each session, participants received stimulation in three different conditions in a pre-defined randomized order. The stimulation conditions targeted either the VMPFC (FpZ), the SPL (Pz), or sham stimulation, which was either delivered over VMPFC or SPL with the coil flipped at 180°. This sham setting gave the participant a similar auditory and tactile experience, while no active stimulation reached the participant’s cortex. Coil positioning and the simulated electric field in each stimulation condition are shown in Figure 1. TMS was applied at 100% individual rMT (mean stimulation intensity = 33.93% (± 3.90 SD) of maximum stimulator output). Individual rMT was defined as the lowest intensity needed to elicit a visible contraction of the left abductor pollicis brevis (APB) in five out of ten pulses after stimulating the right motor cortex.

The stimulation protocol used in all conditions was continuous theta-burst stimulation (cTBS composed of a continuous 40-second train with 600 pulses divided in 3 short bursts of 50 Hz and repeated at a theta range (5 Hz). The stimulation was delivered using a MagVenture ×100 stimulator (MagVenture A/S, Farum, Denmark) and a double cone coil (MagVenture Cool D-B80 MagVenture A/S, Farum, Denmark), which allows deeper cortical stimulation (Cho et al., 2015; Dantas et al., 2023).

#### Heart rate

HR measurements were performed due to the interconnection between HR and vagal tone, which is one of the possible pathways via which probiotic-induced changes are transmitted to the CNS.^63^ HR was measured using a Polar H10 chest strap device (Polar Electro Oy, Kempele, Finland) paired with the recommended Polar Beat software (Polar Electro Oy, Kempele, Finland).^43^ Measurements were taken for 3 minutes before (time 1) and after (time 2) both the task and stimulation at each repetition of the task, resulting in 6 measurements per session and 12 in total. Each HR observation corresponds to the average HR recorded during the 3-minute period.

#### Scales

Several scales were used to control for potential confounding factors. We used the SAM to control for potential effects on mood and arousal^35^ and the BSCS^36^ to measure self-control. A brief dietary questionnaire was administered to control for changes in diet composition,^13^ and finally, we used sections of the Global Preferences Survey (GPS) to evaluate participants’ time and risk preferences.^37^ Our participants were English-speaking residents in the Netherlands, and therefore, the GPS was administered in English, using the validated monetary values of the Dutch version for greater validity. The scales were applied at the beginning of each experimental session.

### Quantification and statistical analysis

#### Risk-taking behavior

Data were pre-processed using custom MATLAB codes. This section analyses participants’ risk taken in the chosen option per trial, which was measured as the chosen option’s payoff standard deviation. For each trial *i*, participants were asked to choose a color that they believed hides the token. Suppose that the probability of a given color option hiding the token is *p* and the payoffs associated with that color *X*. If the participant guesses the color of the box hiding the token correctly (the probability of which is *p*), he can win a reward *X*=*x*, and otherwise he receives *X*=0. Hence, the expected value of this specific trial *i* is *E*(*X*_*i*_)=*xp*. The calculation of this participant’s risk taken in trial *i* is based on standard deviation, or the square root of the trial’s payoff variance, where the variance of payoffs from choosing a color in trial *i* is given by:

(Equation 1) $$Var\left(X_{i}\right)={p\left(x-E\left(X_{i}\right)\right)}^{2}+\left(1-p\right){\left(0-E\left(X_{i}\right)\right)}^{2}$$

The square root of the trial’s variance results in a score of risk-taking behavior (e.g., Myerson (2005), which is the main dependent variable (hereafter, “Risk”).

(Equation 2) $${Risk}_{i}={SD}_{i}=\sqrt{Var\left(X_{i}\right)}$$

Since trials were repeated twice in different order in each repetition of the MGT, participants’ risk-taking behavior was averaged by each trial type (*Risk*_*i*_= (*Risk*_*i*1_+*Risk*_*i*2_)/2), where *i* indexes the 125 unique trials per participant per stimulation condition. This procedure resulted in 125 observations per repetition of the task. The task is repeated once per condition, resulting in 6 repetitions (one for each of the three stimulation conditions (sham, VMPFC, and SPL) and 1 repetition per session (sessions 1 and 2).

Risk was compared across conditions and groups. For instance, when Risk is higher in the probiotics group than in the placebo group, in a specific stimulation condition (sham stimulation, for example) for a specific session (session 2, for example), this means that participants in session 2 with sham stimulation chose riskier options under probiotics than under placebo. Hence, higher values of Risk mean that participants took more risk.

Risk was then analyzed by fitting a linear mixed model (LMM) to predict risk-taking behavior (Risk), with session (sessions 1 and 2), group (probiotics or placebo), stimulation (sham, SPL, and VMPFC), and their interactions (session x group, session x stimulation, stimulation x group, and session x group x stimulation) as factors (formula: Risk ∼ session + group + stimulation + session x group + session x stimulation + stimulation x group + session x group x stimulation), estimated using maximum likelihood (ML) and nloptwarp optimizer, with Bonferroni correction for multiple comparisons. The model included participant and trial type as random effects to account for individual differences in participant’s responses to the different trial types in the MGT. This model was also tested including gender as a main factor. Nevertheless, this addition did not significantly improve the model’s fit and there were no significant differences between genders (p > 05). Hence, gender was removed from the final model.

The final model selection was based on comparison of Akaike Information Criterion (AIC) and Bayesian Information Criterion (BIC) with alternative models including task repetition, gender, and age as fixed factors, and potential covariance structures for the fixed effects. The final analyses presented normally distributed residuals and did not show heteroskedasticity. Due to technical problems, data from 5 repetitions (of different participants) were not properly recorded, resulting in 44375 observations instead of the planned 45000 observations. However, we used LMM in our group-level analyses, which are robust for dealing with missing data.

Considering our partial replication of the TMS methodology used in Dantas et al. (2023), where a significant effect of task repetition on risk-taking behavior was observed, we conducted post hoc analyses including task repetition as a fixed factor.^27^ Our research design included three repetitions of the task per session, named repetitions A, B, and C, respectively. This analysis resulted in an LMM to predict risk-taking behavior (Risk), with session (sessions 1 and 2), group (probiotics or placebo), stimulation (sham, SPL, and VMPFC), repetition (A, B, and C), and their interactions as factors (formula: Risk ∼ session + group + stimulation + repetition + session x group + session x stimulation + stimulation x group + session x repetition + group x repetition + stimulation x repetition + session x group x repetition + session x stimulation x repetition + group x stimulation x repetition + session x group x stimulation + session x group x stimulation x repetition). The model included participant per trial as random effects and was estimated using ML and nloptwarp optimizer. Again, gender was tested as main factor in this LMM and since this addition did not significantly improve the model’s fit and there were no significant differences between genders (p > 05), gender was removed from the final model. The final model selection was based on AIC and BIC, again presenting normally distributed residuals and no heteroskedasticity.

#### Choice optimality

To evaluate participants’ choice optimality (CO), we created a binary score indicating if the participant chose the higher expected value (E) option between the two options in each trial, where 1 indicates the choice of the higher expected value and 0 (zero) indicates otherwise. Since each trial was repeated twice, these scores (0 or 1) were averaged across the repetitions of each unique trial (resulting scores are 0, 0.5 or 1). Afterwards, the effects of each experimental manipulation were estimated by fitting an LMM to predict CO, with session (sessions 1 and 2), group (probiotics or placebo), stimulation (sham, SPL, and VMPFC), and their interactions (session x group, session x stimulation, stimulation x group and session x group x stimulation) as factors (formula: CO ∼ session + group + stimulation + session x group + session x stimulation + stimulation x group + session x group x stimulation). The model again included participant per trial as random effects, was estimated using ML and nloptwarp optimizer, with Bonferroni correction for multiple comparisons. Gender was also tested as main effect in this LMM, and since its addition did not improve the model’s fit nor yielded significant results, it was removed from the final model. The final model selection was based on AIC and BIC.

#### Response time

We calculated response time (RT) using the difference in seconds from the start of the trial and the participants’ finger-press response on the keyboard. Response time was also averaged across the same type of trial, resulting in 125 observations in each repetition of the game. Outlier correction was needed for this variable, which was done using custom R scripts to remove observations outside 1.5 times the interquartile range.^64^ A total of 988 observations (of different participants) were removed, leaving 43387 observations.

Group analyses were then performed by fitting an LMM to predict RT, with session (sessions 1 and 2), group (probiotics or placebo), stimulation (sham, SPL, and VMPFC), and their interactions (session x group, session x stimulation, stimulation x group and session x group x stimulation) as factors (formula: RT ∼ session + group + stimulation + session x group + session x stimulation + stimulation x group + session x group x stimulation, and estimated using maximum likelihood (ML) and nloptwarp optimizer), with Bonferroni correction for multiple comparisons. Gender was tested as a main factor, and the model including gender yielded significantly lower AIC and BIC, with male participants displaying significantly higher response times (beta = 0.22, 95% CI [0.05, 0.40], t(43360) = 2.54, p = 0.011) than females. Yet, for the sake of consistency and considering that response times are a secondary dependent variable in our study, we opted for using a final LMM with the same main factors used to evaluate Risk-taking behavior and choice optimality. The final model selection was then based on AIC and BIC, which presented normally distributed residuals and no heteroskedasticity.

#### Heart rate

For the heart rate (HR) analyses, we averaged participants’ HR during the 3 minutes measured before and after each stimulation application (3 rounds, one for each stimulation condition). The data was then analyzed by fitting an LMM (estimated using REML and nloptwrap optimizer) to predict HR with session (sessions 1 and 2), group (probiotics and placebo), stimulation (VMPFC, SPL, and sham), and time (before and after stimulation) and its interactions as fixed factors. The model included participant as random effect. For parsimony, nonsignificant interactions that were not necessary for the hypotheses testing were removed from the model (formula: HR ∼ session + group + stimulation + time + session x group).

#### Scales

All scale results were analyzed using LMMs with session (sessions 1 and 2), group (probiotics and placebo), and the interaction session x group as fixed factors. All models were estimated using REML and nloptwrap optimizer, with Bonferroni corrections for multiple comparisons.
