# Supplementary material for: A gut feeling: Exploring the effects of probiotics on risk-taking behavior using TMS
Source: iScience. 2026 Jan 14;29(2):114696. doi: 10.1016/j.isci.2026.114696 (PMC12907717; doi:10.1016/j.isci.2026.114696)
Supplement: Document S1. Tables S1–S13 [file mmc1.pdf]

iScience, Volume *29*

Supplemental information

A gut feeling: Exploring the effects of probiotics on risk-taking behavior using TMS

Aline M. Dantas, Teresa Schuhmann, Elisabeth Brüggen, and Peiran Jiao

Supporting information

Table S1. Risk-taking behavior post hoc results, related to Figure 1: Fixed effects from the post hoc LMM (formula: RISK ~ SESSION + GROUP + STIMULATION + REPETITION + SESSION * GROUP + SESSION * STIMULATION + STIMULATION * GROUP + SESSION * REPETITION + GROUP * REPETITION + STIMULATION * REPETITION + SESSION * GROUP * REPETITION + SESSION * STIMULATION * REPETITION + GROUP * STIMULATION * REPETITION + SESSION * GROUP * STIMULATION + SESSION * GROUP * STIMULATION * REPETITION). The model included TRIALCODE as random effects (formula: list(~1 | TRIALCODE:PARTICIPANT, ~1 | PARTICIPANT)).

	**Risk-taking behavior**				
Predictors	*Estimates*	*std. Error*	*CI*	*p*	*df*
(Intercept)	25.4	0.36	24.71 – 26.10	**<0.001**	44336
SESSION [2]	0.04	0.19	-0.33 – 0.41	0.832	44336
GROUP [PROBIOTICS]	0.87	0.49	-0.09 – 1.83	0.077	44336
STIMULATION [SPL]	-0.19	0.2	-0.58 – 0.19	0.329	44336
STIMULATION [VMPFC]	0.52	0.18	0.17 – 0.87	**0.004**	44336
REPETITION [B]	0.21	0.21	-0.19 – 0.62	0.302	44336
REPETITION [C]	-0.31	0.17	-0.66 – 0.03	0.072	44336
SESSION [2] × GROUP [PROBIOTICS]	0.59	0.25	0.09 – 1.09	**0.02**	44336
SESSION [2] × STIMULATION [SPL]	-0.24	0.29	-0.80 – 0.33	0.41	44336
SESSION [2] × STIMULATION [VMPFC]	-0.69	0.26	-1.21 – -0.17	**0.009**	44336
GROUP [PROBIOTICS] × STIMULATION [SPL]	0.82	0.26	0.31 – 1.34	**0.002**	44336
GROUP [PROBIOTICS] × STIMULATION [VMPFC]	-0.45	0.26	-0.96 – 0.06	0.085	44336
SESSION [2] × REPETITION [B]	-0.23	0.28	-0.78 – 0.32	0.409	44336
SESSION [2] × REPETITION [C]	-0.01	0.27	-0.54 – 0.53	0.985	44336
GROUP [PROBIOTICS] × REPETITION [B]	0.39	0.27	-0.14 – 0.92	0.147	44336
GROUP [PROBIOTICS] × REPETITION [C]	0.73	0.26	0.23 – 1.24	**0.004**	44336
STIMULATION [SPL] × REPETITION [B]	-0.11	0.3	-0.69 – 0.48	0.723	44336
STIMULATION [VMPFC] × REPETITION [B]	-1.18	0.28	-1.74 – -0.63	**<0.001**	44336
STIMULATION [SPL] × REPETITION [C]	0.44	0.28	-0.10 – 0.98	0.114	44336
STIMULATION [VMPFC] × REPETITION [C]	-0.06	0.29	-0.64 – 0.51	0.834	44336
(SESSION [2] × GROUP	-0.54	0.37	-1.27 – 0.19	0.15	44336

[PROBIOTICS]) × REPETITION [B]					
(SESSION [2] × GROUP [PROBIOTICS]) × REPETITION [C]	-0.22	0.38	-0.96 – 0.52	0.56	44336
(SESSION [2] × STIMULATION [SPL]) × REPETITION [B]	0.42	0.41	-0.39 – 1.22	0.31	44336
(SESSION [2] × STIMULATION [VMPFC]) × REPETITION [B]	1.05	0.4	0.26 – 1.83	**0.009**	44336
(SESSION [2] × STIMULATION [SPL]) × REPETITION [C]	-0.32	0.41	-1.13 – 0.49	0.443	44336
(SESSION [2] × STIMULATION [VMPFC]) × REPETITION [C]	0.33	0.42	-0.49 – 1.15	0.426	44336
(GROUP [PROBIOTICS] × STIMULATION [SPL]) × REPETITION [B]	-0.61	0.39	-1.38 – 0.16	0.12	44336
(GROUP [PROBIOTICS] × STIMULATION [VMPFC]) × REPETITION [B]	1.06	0.39	0.29 – 1.83	**0.007**	44336
(GROUP [PROBIOTICS] × STIMULATION [SPL]) × REPETITION [C]	-1.3	0.39	-2.07 – -0.53	**0.001**	44336
(GROUP [PROBIOTICS] × STIMULATION [VMPFC]) × REPETITION [C]	-0.1	0.4	-0.88 – 0.68	0.804	44336
(SESSION [2] × GROUP [PROBIOTICS]) × STIMULATION [SPL]	-0.65	0.38	-1.39 – 0.10	0.088	44336
(SESSION [2] × GROUP [PROBIOTICS]) × STIMULATION [VMPFC]	0.31	0.37	-0.42 – 1.04	0.404	44336
(SESSION [2] × GROUP [PROBIOTICS] × STIMULATION [SPL]) × REPETITION [B]	0.82	0.55	-0.25 – 1.90	0.133	44336
(SESSION [2] × GROUP [PROBIOTICS] × STIMULATION [VMPFC]) × REPETITION [B]	-0.72	0.55	-1.81 – 0.36	0.192	44336
(SESSION [2] × GROUP [PROBIOTICS] × STIMULATION [SPL]) × REPETITION [C]	1.29	0.57	0.18 – 2.40	**0.023**	44336
(SESSION [2] × GROUP [PROBIOTICS] × STIMULATION [VMPFC]) × REPETITION [C]	-0.3	0.57	-1.41 – 0.81	0.596	44336
**Random Effects**					
σ^2	16.84				
τ_{00} TRIALCODE:PARTICIPANT	193				

τ_{00} PARTICIPANT	1.6
ICC	0.92
N TRIALCODE	125
N PARTICIPANT	60
Observations	44375
Marginal R^2 / Conditional R^2	0.003 / 0.921
AIC	283140.546

Table S2. Risk-taking behavior post hoc results, related to Figure 1: Contrasts analyses - group differences by stimulation condition and session.

Risk-taking behavior

GROUP	*STIMULATION*	*REPETITION*	*SESSION*	*contrast*	*estimate*	*SE*	*z.ratio*	*p.value*
PLACEBO	SHAM	A	.	SESSION2 - SESSION1	0.0398	0.187	0.213	1
PROBIOTICS	SHAM	A	.	SESSION2 - SESSION1	0.6322	0.173	3.659	**0.0172**
PLACEBO	SPL	A	.	SESSION2 - SESSION1	-0.1968	0.205	-0.959	1
PROBIOTICS	SPL	A	.	SESSION2 - SESSION1	-0.2497	0.164	-1.519	0.9967
PLACEBO	VMPFC	A	.	SESSION2 - SESSION1	-0.6503	0.169	-3.851	0.0081
PROBIOTICS	VMPFC	A	.	SESSION2 - SESSION1	0.2531	0.188	1.344	0.9996
PLACEBO	SHAM	B	.	SESSION2 - SESSION1	-0.1921	0.202	-0.952	1
PROBIOTICS	SHAM	B	.	SESSION2 - SESSION1	-0.1387	0.169	-0.823	1
PLACEBO	SPL	B	.	SESSION2 - SESSION1	-0.0123	0.198	-0.062	1
PROBIOTICS	SPL	B	.	SESSION2 - SESSION1	0.2195	0.162	1.356	0.9995
PLACEBO	VMPFC	B	.	SESSION2 - SESSION1	0.1662	0.169	0.986	1
PROBIOTICS	VMPFC	B	.	SESSION2 - SESSION1	-0.1938	0.204	-0.952	1
PLACEBO	SHAM	C	.	SESSION2 - SESSION1	0.0347	0.175	0.199	1
PROBIOTICS	SHAM	C	.	SESSION2 - SESSION1	0.4075	0.184	2.209	0.7415
PLACEBO	SPL	C	.	SESSION2 - SESSION1	-0.5199	0.165	-3.158	0.0941
PROBIOTICS	SPL	C	.	SESSION2 - SESSION1	0.4973	0.205	2.427	0.5508
PLACEBO	VMPFC	C	.	SESSION2 - SESSION1	-0.322	0.242	-1.333	0.9997
PROBIOTICS	VMPFC	C	.	SESSION2 - SESSION1	0.0613	0.148	0.414	1
.	SHAM	A	1	PROBIOTICS - PLACEBO	0.8699	0.492	1.769	0.9706
.	SHAM	A	2	PROBIOTICS - PLACEBO	1.4623	0.493	2.967	0.1624
.	SPL	A	1	PROBIOTICS - PLACEBO	1.6909	0.494	3.425	**0.0388**
.	SPL	A	2	PROBIOTICS - PLACEBO	1.638	0.496	3.304	0.0571
.	VMPFC	A	1	PROBIOTICS - PLACEBO	0.4191	0.493	0.85	1
.	VMPFC	A	2	PROBIOTICS - PLACEBO	1.3226	0.49	2.696	0.3254

.	SHAM	B	1	PROBIOTICS - PLACEBO	1.2628	0.499	2.533	0.4545
.	SHAM	B	2	PROBIOTICS - PLACEBO	1.3162	0.493	2.668	0.3467
.	SPL	B	1	PROBIOTICS - PLACEBO	1.4735	0.492	2.994	0.1523
.	SPL	B	2	PROBIOTICS - PLACEBO	1.7053	0.492	3.468	**0.0331**
.	VMPFC	B	1	PROBIOTICS - PLACEBO	1.8731	0.492	3.805	**0.0096**
.	VMPFC	B	2	PROBIOTICS - PLACEBO	1.5131	0.496	3.053	0.1292
.	SHAM	C	1	PROBIOTICS - PLACEBO	1.602	0.49	3.268	0.0649
.	SHAM	C	2	PROBIOTICS - PLACEBO	1.9748	0.493	4.003	**0.0046**
.	SPL	C	1	PROBIOTICS - PLACEBO	1.1235	0.496	2.266	0.6949
.	SPL	C	2	PROBIOTICS - PLACEBO	2.1407	0.493	4.343	0.0011
.	VMPFC	C	1	PROBIOTICS - PLACEBO	1.0526	0.502	2.095	0.8275
.	VMPFC	C	2	PROBIOTICS - PLACEBO	1.4359	0.498	2.881	0.2068
PLACEBO	.	A	1	SPL - SHAM	-0.1915	0.196	-0.976	1
PLACEBO	.	A	1	VMPFC - SPL	0.7123	0.2	3.555	**0.0248**
PLACEBO	.	A	2	SPL - SHAM	-0.428	0.205	-2.091	0.831
PLACEBO	.	A	2	VMPFC - SPL	0.2587	0.186	1.391	0.9993
PROBIOTICS	.	A	1	SPL - SHAM	0.6295	0.175	3.597	**0.0216**
PROBIOTICS	.	A	1	VMPFC - SPL	-0.5595	0.174	-3.207	0.0799
PROBIOTICS	.	A	2	SPL - SHAM	-0.2524	0.175	-1.445	0.9986
PROBIOTICS	.	A	2	VMPFC - SPL	-0.0567	0.188	-0.302	1
PLACEBO	.	B	1	SPL - SHAM	-0.2971	0.215	-1.383	0.9994
PLACEBO	.	B	1	VMPFC - SPL	-0.3637	0.18	-2.026	0.8706
PLACEBO	.	B	2	SPL - SHAM	-0.1173	0.193	-0.607	1
PLACEBO	.	B	2	VMPFC - SPL	-0.1852	0.194	-0.956	1
PROBIOTICS	.	B	1	SPL - SHAM	-0.0863	0.167	-0.518	1
PROBIOTICS	.	B	1	VMPFC - SPL	0.0358	0.191	0.187	1
PROBIOTICS	.	B	2	SPL - SHAM	0.2718	0.177	1.535	0.9962
PROBIOTICS	.	B	2	VMPFC - SPL	-0.3774	0.183	-2.061	0.8502
PLACEBO	.	C	1	SPL - SHAM	0.244	0.172	1.422	0.9989
PLACEBO	.	C	1	VMPFC - SPL	0.2152	0.217	0.993	1
PLACEBO	.	C	2	SPL - SHAM	-0.3106	0.176	-1.761	0.9722
PLACEBO	.	C	2	VMPFC - SPL	0.4131	0.204	2.03	0.8687
PROBIOTICS	.	C	1	SPL - SHAM	-0.2344	0.201	-1.166	1
PROBIOTICS	.	C	1	VMPFC - SPL	0.1443	0.186	0.775	1
PROBIOTICS	.	C	2	SPL - SHAM	-0.1446	0.197	-0.733	1
PROBIOTICS	.	C	2	VMPFC - SPL	-0.2918	0.183	-1.593	0.993
PLACEBO	SHAM	.	1	B - A	0.2123	0.206	1.031	1
PLACEBO	SHAM	.	1	C - B	-0.527	0.207	-2.551	0.441
PLACEBO	SHAM	.	2	B - A	-0.0196	0.193	-0.102	1
PLACEBO	SHAM	.	2	C - B	-0.3001	0.182	-1.646	0.9887
PROBIOTICS	SHAM	.	1	B - A	0.6051	0.177	3.426	**0.0384**
PROBIOTICS	SHAM	.	1	C - B	-0.1878	0.173	-1.087	1

PROBIOTICS	SHAM	.	2	B - A	-0.1658	0.18	-0.922	1
PROBIOTICS	SHAM	.	2	C - B	0.3585	0.192	1.867	0.9433
PLACEBO	SPL	.	1	B - A	0.1066	0.206	0.519	1
PLACEBO	SPL	.	1	C - B	0.0141	0.182	0.078	1
PLACEBO	SPL	.	2	B - A	0.2911	0.203	1.432	0.9988
PLACEBO	SPL	.	2	C - B	-0.4934	0.187	-2.636	0.3706
PROBIOTICS	SPL	.	1	B - A	-0.1107	0.165	-0.671	1
PROBIOTICS	SPL	.	1	C - B	-0.3359	0.196	-1.713	0.9807
PROBIOTICS	SPL	.	2	B - A	0.3584	0.172	2.086	0.8344
PROBIOTICS	SPL	.	2	C - B	-0.058	0.183	-0.317	1
PLACEBO	VMPFC	.	1	B - A	-0.9694	0.173	-5.606	**<.0001**
PLACEBO	VMPFC	.	1	C - B	0.5931	0.215	2.761	0.2828
PLACEBO	VMPFC	.	2	B - A	-0.1528	0.175	-0.874	1
PLACEBO	VMPFC	.	2	C - B	0.1049	0.21	0.501	1
PROBIOTICS	VMPFC	.	1	B - A	0.4846	0.199	2.433	0.5451
PROBIOTICS	VMPFC	.	1	C - B	-0.2274	0.181	-1.258	0.9999
PROBIOTICS	VMPFC	.	2	B - A	0.0377	0.198	0.191	1
PROBIOTICS	VMPFC	.	2	C - B	0.0276	0.183	0.151	1

Table S3. Choice Optimality results, related to STARMethods section QUANTIFICATION AND STATISTICAL ANALYSIS - Choice Optimality: Fixed effects from the primary LMM (formula: ChoiceOptimality ~ SESSION + GROUP + CONDITION + SESSION * GROUP + SESSION * CONDITION + CONDITION * GROUP + SESSION * GROUP * CONDITION). The model included TRIALCODE as random effects (formula: list(~1 | TRIALCODE:PARTICIPANT, ~1 | PARTICIPANT)).

	Choice Optimality				
Predictors	*Estimates*	*std. Error*	*CI*	*p*	*df*
(Intercept)	0.85	0.01	0.83 – 0.88	**<0.001**	44360
SESSION [2]	0	0	-0.01 – 0.00	0.428	44360
GROUP [PROBIOTICS]	0.03	0.02	0.00 – 0.06	**0.025**	44360
STIMULATION [SPL]	0	0	-0.01 – 0.01	0.863	44360
STIMULATION [VMPFC]	0.01	0	-0.00 – 0.01	0.098	44360
SESSION [2] × GROUP [PROBIOTICS]	0	0.01	-0.01 – 0.01	0.855	44360
SESSION [2] × STIMULATION [SPL]	-0.01	0.01	-0.02 – 0.00	0.083	44360
SESSION [2] × STIMULATION [VMPFC]	0	0.01	-0.02 – 0.01	0.407	44360
GROUP [PROBIOTICS] × STIMULATION [SPL]	0	0.01	-0.01 – 0.01	0.64	44360
GROUP [PROBIOTICS] × STIMULATION [VMPFC]	-0.01	0.01	-0.02 – -0.00	**0.049**	44360
(SESSION [2] × GROUP [PROBIOTICS]) × STIMULATION [SPL]	0.01	0.01	-0.01 – 0.02	0.515	44360
(SESSION [2] × GROUP [PROBIOTICS]) × STIMULATION [VMPFC]	0	0.01	-0.02 – 0.02	0.96	44360
**Random Effects**					
σ^2	0.03				
τ_{00} TRIALCODE:PARTICIPANT	0.05				
τ_{00} PARTICIPANT	0				
ICC	0.66				
N TRIALCODE	125				
N PARTICIPANT	60				
Observations	44375				
Marginal R^2 / Conditional R^2	0.003 / 0.658				
AIC	-10879.53				

Table S4. Choice Optimality results, related STARMethods section QUANTIFICATION AND STATISTICAL ANALYSIS - Choice Optimality: Contrasts analyses (post hoc LMM) - group differences by stimulation condition and session.

Choice Optimality							
GROUP	*STIMULATION*	*SESSION*	*contrast*	*estimate*	*SE*	*z.ratio*	*p.value*
PLACEBO	SHAM	.	SESSION2 - SESSION1	-0.00329	0.00414	-0.793	0.9982
PROBIOTICS	SHAM	.	SESSION2 - SESSION1	-0.00224	0.00396	-0.565	0.9999
PLACEBO	SPL	.	SESSION2 - SESSION1	-0.01343	0.00414	-3.241	0.0185
PROBIOTICS	SPL	.	SESSION2 - SESSION1	-0.00712	0.00392	-1.819	0.6006
PLACEBO	VMPFC	.	SESSION2 - SESSION1	-0.00814	0.00414	-1.966	0.4861
PROBIOTICS	VMPFC	.	SESSION2 - SESSION1	-0.00669	0.00396	-1.692	0.6981
.	SHAM	1	PROBIOTICS - PLACEBO	0.033902	0.01509	2.247	0.2908
.	SHAM	2	PROBIOTICS - PLACEBO	0.034951	0.01511	2.313	0.2521
.	SPL	1	PROBIOTICS - PLACEBO	0.036554	0.01509	2.423	0.1974
.	SPL	2	PROBIOTICS - PLACEBO	0.042859	0.0151	2.838	0.0657
.	VMPFC	1	PROBIOTICS - PLACEBO	0.022732	0.01509	1.507	0.8237
.	VMPFC	2	PROBIOTICS - PLACEBO	0.024182	0.01511	1.6	0.7634
PLACEBO	.	1	SPL - SHAM	-0.00071	0.00414	-0.172	1
PLACEBO	.	1	VMPFC - SPL	0.007571	0.00414	1.828	0.5935
PLACEBO	.	2	SPL - SHAM	-0.01086	0.00414	-2.621	0.1209
PLACEBO	.	2	VMPFC - SPL	0.012857	0.00414	3.104	0.0293
PROBIOTICS	.	1	SPL - SHAM	0.001937	0.00388	0.5	1
PROBIOTICS	.	1	VMPFC - SPL	-0.00625	0.00388	-1.613	0.7548
PROBIOTICS	.	2	SPL - SHAM	-0.00295	0.00398	-0.742	0.999
PROBIOTICS	.	2	VMPFC - SPL	-0.00582	0.00398	-1.464	0.8484

Table S5. Heart rate – Results, related STARMethods section QUANTIFICATION AND STATISTICAL ANALYSIS – Heart Rate

	Heart rate				
Predictors	*Estimates*	*std. Error*	*CI*	*p*	*df*
(Intercept)	74.55	2.2	70.23 – 78.86	**<0.001**	656
SESSION	-0.12	0.76	-1.61 – 1.36	0.869	656
GROUP[PROBIOTICS]	1.2	2.91	-4.52 – 6.92	0.681	656
STIMULATION [SPL]	-0.57	0.62	-1.79 – 0.65	0.361	656
STIMULATION [VMPFC]	-1.05	0.62	-2.27 – 0.17	0.092	656
TIME	-1.48	0.51	-2.47 – -0.49	**0.003**	656
SESSION × GROUP [PROBIOTICS]	0.27	1.04	-1.77 – 2.30	0.796	656
**Random Effects**					
σ^2	42.4				
$\tau_{00\ \text{Participant}}$	86.37				
ICC	0.67				
$N_{\text{Participant}}$	60				
Observations	665				
Marginal R^2 / Conditional R^2	0.011 / 0.674				
AIC	4568.429				

Table S6. Control scales: Dietary scale – Results, related STARMethods section METHOD DETAILS - Scales

	**Diet**				
Predictors	*Estimates*	*std. Error*	*CI*	*p*	*df*
(Intercept)	26.85	3.98	18.97 – 34.73	**<0.001**	111
SESSION	-2.64	2.54	-7.67 – 2.39	0.301	111
GROUP [PROBIOTICS]	-5.32	4.92	-15.06 – 4.43	0.282	111
SESSION × GROUP [PROBIOTICS]	2.15	3.12	-4.04 – 8.34	0.49	111
**Random Effects**					
σ^2	70.82				
τ_{00} SESSION	0.6				
ICC	0.01				
N SESSION	2				
Observations	117				
Marginal R^2 / Conditional R^2	0.027 / 0.035				
AIC	827.565				

Table S7. Control scales: BSCS – Results, related STARMethods section METHOD DETAILS - Scales

	BSCS				
Predictors	*Estimates*	*std. Error*	*CI*	*p*	*df*
(Intercept)	41.77	3.57	34.71 – 48.84	**<0.001**	111
SESSION	-0.56	2.28	-5.08 – 3.96	0.806	111
GROUP [PROBIOTICS]	-1.02	4.78	-10.49 – 8.44	0.83	111
SESSION × GROUP [PROBIOTICS]	-0.19	3.03	-6.20 – 5.82	0.95	111
**Random Effects**					
σ^2	66.75				
τ_{00} SESSION	0.12				
ICC	0				
N SESSION	2				
Observations	117				
Marginal R^2 / Conditional R^2	0.008 / 0.010				
AIC	820.876				

Table S8. Control scales: SAM (Mood) – Results, related STARMethods section METHOD DETAILS - Scales

	SAM - Mood				
Predictors	*Estimates*	*std. Error*	*CI*	*p*	*df*
(Intercept)	3.69	0.22	3.24 – 4.13	**<0.001**	111
SESSION	0.1	0.14	-0.19 – 0.38	0.493	111
GROUP [PROBIOTICS]	0.34	0.31	-0.27 – 0.95	0.272	111
SESSION × GROUP [PROBIOTICS]	-0.19	0.2	-0.58 – 0.20	0.33	111
**Random Effects**					
σ^2	0.28				
τ_{00} SESSION	0				
ICC	0				
N SESSION	2				
Observations	117				
Marginal R^2 / Conditional R^2	0.011 / 0.011				
AIC	201.673				

Table S9. Control scales: SAM (Arousal) – Results, related STARMethods section METHOD DETAILS - Scales

	SAM - Arousal				
Predictors	*Estimates*	*std. Error*	*CI*	*p*	*df*
(Intercept)	2.8	0.39	2.03 – 3.57	**<0.001**	111
SESSION	-0.27	0.25	-0.76 – 0.22	0.285	111
GROUP [PROBIOTICS]	-0.54	0.47	-1.47 – 0.39	0.251	111
SESSION × GROUP [PROBIOTICS]	0.2	0.3	-0.39 – 0.79	0.51	111
**Random Effects**					
σ^2	0.64				
τ_{00} SESSION	0.01				
ICC	0.01				
N SESSION	2				
Observations	117				
Marginal R^2 / Conditional R^2	0.036 / 0.047				
AIC	295.893				

Table S10. Control scales: GPS (Qualitative evaluation of Risk) – Results, related STARMethods section METHOD DETAILS - Scales

	GPS – Qualitative evaluation of Risk				
Predictors	*Estimates*	*std. Error*	*CI*	*p*	*df*
(Intercept)	4.58	0.9	2.80 – 6.36	**<0.001**	111
SESSION	0.67	0.57	-0.46 – 1.81	0.244	111
GROUP [PROBIOTICS]	1.43	1.2	-0.94 – 3.80	0.233	111
SESSION × GROUP [PROBIOTICS]	-1.04	0.76	-2.54 – 0.47	0.18	111
**Random Effects**					
σ^2	4.18				
τ_{00} SESSION	0.01				
ICC	0				
N SESSION	2				
Observations	117				
Marginal R^2 / Conditional R^2	0.017 / 0.020				
AIC	507.872				

Table S11. Control scales: GPS (Quantitative evaluation of Risk) – Results, related STARMethods section METHOD DETAILS - Scales

	GPS – Implied switching row (Risk quantitative)				
Predictors	*Estimates*	*std. Error*	*CI*	*p*	*df*
(Intercept)	8.19	2.17	3.88 – 12.50	**<0.001**	109
SESSION	1.31	1.39	-1.45 – 4.06	0.349	109
GROUP [PROBIOTICS]	4.59	2.93	-1.23 – 10.40	0.121	109
SESSION × GROUP [PROBIOTICS]	-1.76	1.87	-5.47 – 1.94	0.35	109
**Random Effects**					
σ^2	25.04				
τ_{00} SESSION	0.04				
ICC	0				
N SESSION	2				
Observations	115				
Marginal R^2 / Conditional R^2	0.046 / 0.047				
AIC	697.896				

Table S12. Control scales: GPS (Qualitative evaluation of Time Preferences) – Results, related STARMethods section METHOD DETAILS - Scales

	GPS – Qualitative Time Preferences				
Predictors	*Estimates*	*std. Error*	*CI*	*p*	*df*
(Intercept)	5.63	0.71	4.22 – 7.04	**<0.001**	111
SESSION	0.41	0.45	-0.49 – 1.31	0.372	111
GROUP [PROBIOTICS]	-0.39	0.84	-2.06 – 1.28	0.641	111
SESSION × GROUP [PROBIOTICS]	-0.13	0.53	-1.19 – 0.93	0.81	111
**Random Effects**					
σ^2	2.08				
$\tau_{00\ SESSION}$	0.03				
ICC	0.01				
N SESSION	2				
Observations	117				
Marginal R^2 / Conditional R^2	0.051 / 0.062				
AIC	428.812				

Table S13. Control scales: GPS (Quantitative evaluation of Time Preferences) – Results, related STARMethods section METHOD DETAILS - Scales

	**GPS – Quantitative Time Preferences**				
Predictors	*Estimates*	*std. Error*	*CI*	*p*	*df*
(Intercept)	20.48	3.98	12.59 – 28.38	**<0.001**	111
SESSION	-0.13	2.55	-5.17 – 4.92	0.961	111
GROUP [PROBIOTICS]	-0.62	5.34	-11.20 – 9.96	0.908	111
SESSION × GROUP [PROBIOTICS]	1.55	3.39	-5.16 – 8.27	0.65	111
**Random Effects**					
σ^2	83.49				
τ_{00} SESSION	0.15				
ICC	0				
N SESSION	2				
Observations	117				
Marginal R^2 / Conditional R^2	0.012 / 0.014				
AIC	846.159				
